# Modeling Polygenic Antibiotic Resistance Evolution in Biofilms

**DOI:** 10.3389/fmicb.2022.916035

**Published:** 2022-07-07

**Authors:** Barbora Trubenová, Dan Roizman, Jens Rolff, Roland R. Regoes

**Affiliations:** ^1^Institute of Integrative Biology, ETH Zürich, Zurich, Switzerland; ^2^Institute of Biology – Evolutionary Biology, Freie Universität Berlin, Berlin, Germany

**Keywords:** biofilm recalcitrance, population genetics, antibiotic resistance, resistance evolution, mathematical modeling, PK/PD

## Abstract

The recalcitrance of biofilms to antimicrobials is a multi-factorial phenomenon, including genetic, physical, and physiological changes. Individually, they often cannot account for biofilm recalcitrance. However, their combination can increase the minimal inhibitory concentration of antibiotics needed to kill bacterial cells by three orders of magnitude, explaining bacterial survival under otherwise lethal drug treatment. The relative contributions of these factors depend on the specific antibiotics, bacterial strain, as well as environmental and growth conditions. An emerging population genetic property—increased biofilm genetic diversity—further enhances biofilm recalcitrance. Here, we develop a polygenic model of biofilm recalcitrance accounting for multiple phenotypic mechanisms proposed to explain biofilm recalcitrance. The model can be used to generate predictions about the emergence of resistance—its timing and population genetic consequences. We use the model to simulate various treatments and experimental setups. Our simulations predict that the evolution of resistance is impaired in biofilms at low antimicrobial concentrations while it is facilitated at higher concentrations. In scenarios that allow bacteria exchange between planktonic and biofilm compartments, the evolution of resistance is further facilitated compared to scenarios without exchange. We compare these predictions to published experimental observations.

## 1. Introduction

Biofilms are heterogeneous communities of bacteria attached to a substrate or each other, forming aggregates that can be visible by the naked eye. Biofilms are very difficult to remove and cause significant problems in many aspects of human lives: from industry and households, where their large colonies block water pipes, to human health, affecting all body systems: they grow on teeth, tongues, eyes and skin, contact lenses, catheters, and medical implants (Donlan, 2002; Ciofu et al., 2017). Mature biofilms can survive in antibiotic concentrations thousands of times higher than those killing planktonic cells (Nickel et al., 1985; Sharma et al., 2019). This ability, denoted *recalcitrance*, allows biofilms to serve as reservoirs of bacterial cells that survive antibiotic treatment, further releasing bacterial cells. Biofilms cause chronic infections in wounds, tooth decay, and can cause tissue damage by eliciting persistent immune responses or even cancer (Ciofu et al., 2022). It has been shown that some *genetic*, heritable mutations conferring antibiotic resistance arise in biofilms (Sharma et al., 2019). These represent a significant problem for health care, as the resistance is retained by bacterial cells upon dispersal, leading to difficulties in subsequent infection treatments and the spread of antibiotic resistance (Jorge et al., 2019).

However, bacterial cells often lose their recalcitrance when they disperse from the biofilm. Therefore, the recalcitrance cannot be attributed solely to mutations and *genetic* changes in biofilm cells. Other, *phenotypic* adaptations of individual cells, as well as the presence of an extracellular matrix, must contribute to biofilm recalcitrance. Numerous explanations for the observed recalcitrance of biofilms have been proposed and thoroughly reviewed (Stewart, 2002; Venkatesan et al., 2015; Ciofu et al., 2017; Hall and Mah, 2017; Hathroubi et al., 2017; Valquier-Flynn et al., 2017; Roy et al., 2018; Crabbé et al., 2019; Gebreyohannes et al., 2019; Sharma et al., 2019; Yan and Bassler, 2019; Bottery et al., 2021). For instance, biofilms can resist penetration by antimicrobials, degrade them by enzymes present in the extracellular matrix, or interact with other extracellular polymeric substances (EPS), such as enzymes, lipids, or extracellular DNA (eDNA); these have been recently shown to provide cooperative fitness to biofilm populations (Belcher et al., 2022). Cells with low metabolic activity offer fewer targets for antimicrobials, rendering many of them ineffective.

It is now widely accepted that multiple genes of various effects determine antibiotic resistance in planktonic bacteria (Petchiappan and Chatterji, 2017; Apjok et al., 2019; Igler et al., 2021). Similarly, multiple mutations were recently implicated in biofilm recalcitrance (Santos-Lopez et al., 2019; Santos-Lopez and Cooper, 2021). Evolutionary experiments have shown that when biofilm and planktonic bacteria are exposed to increasing concentrations of antibiotics, the biofilm populations harbor even higher genetic diversity than planktonic populations that experienced the same treatment (Ahmed et al., 2018, 2020; Santos-Lopez et al., 2019; Santos-Lopez and Cooper, 2021).

Understanding interactions between the phenotypic and genotypic factors influencing biofilm recalcitrance is crucial for maximizing the probability of successful treatment and minimizing the risk of antibiotic resistance evolution. These interactions and their consequences have recently been discussed in Trubenová et al. (2022). We reasoned that the effects of individual recalcitrance mechanisms combine in non-intuitive ways and can either hinder or promote resistance evolution, depending mainly on the concentration of the antibiotics. Building on these verbal arguments and hypotheses, we here provide a quantitative modeling approach, investigating the role of the different drivers of biofilm recalcitrance.

Despite the fact that the biofilm is a predominant bacterial lifestyle, most experiments are performed with planktonic bacteria. Similarly, models of antibiotic resistance evolution are abundant. However, mathematical models and simulations of biofilm growth often focus on the formation of the spatial biofilm structure, modeling physical and chemical processes such as cell and nutrient transport, metabolic reaction, hydrodynamics, biomass growth, and detachment (Picioreanu et al., 2007; Kragh et al., 2016; Ali and Wahl, 2017; Brockmann et al., 2020). For instance, Stewart (2003), Stewart et al. (2016) modeled diffusion in biofilms, while Picioreanu et al. (2007) modeled 3D structure of *Pseudomonas aeruginosa* biofilms, that form mushroom-like colonies. These models and predictions are useful for industry, where biofilm mass, shape, and other physical properties are important. They typically do not deal with multiple strains and the possibility of resistance mutations. Only a minority of models focuses on the population dynamic and genetic aspects that are central to the evolution and spread of resistance in biofilms (Torella et al., 2010; Eastman et al., 2011; Raynes et al., 2018).

Here, we develop a polygenic model of biofilm recalcitrance that allows us to simultaneously study the main phenotypic recalcitrance mechanisms proposed in the literature: (a) those relying on extracellular polymeric substances (EPS, such as extracellular DNA, enzymes, or lipids) acting as a physical barrier, reducing the antibiotic concentration that bacteria experience inside the biofilm; and (b) those relying on physiological alterations, such as slow replication or metabolism, or high fraction of persisters. We will use a pharmacodynamic modeling framework to capture both the genetic and phenotypic mechanisms acting in biofilms, as discussed in Trubenová et al. (2022), and derive population replication, death, and mutation rates in the presence of antibiotics. The main question we address is under which conditions biofilms accelerate or delay antibiotic resistance evolution. Based on arguments discussed in the literature (Trubenová et al., 2022, and references therein), the biofilm lifestyle is expected to reduce selection pressure for mutations at low concentrations, thus slowing down the evolution of resistance. By contrast, it is expected to enable the evolution of resistance under higher concentrations that kill the free-living bacteria. Here, we will show under which conditions these arguments hold and what other factors influence biofilm survival and resistance evolution.

## 2. The Model

Our simulation model uses a framework we proposed in Trubenová et al. (2022) that extends pharmaco-kinetic and -dynamic models by multiple bacterial genotypes with varying degrees of resistance to the antimicrobial. Planktonic and biofilm lifestyles are captured by differences in the bacterial replication rates and the efficacy of antimicrobials. We ignore the spatial aspects of biofilm formation and growth. In most of our analysis, we compare the evolutionary dynamics in systems that adopt either a completely planktonic lifestyle or live exclusively as a biofilm. Nevertheless, we also consider a model variant with both lifestyles and investigate how the exchange affects resistance evolution.

**Figure 1:**
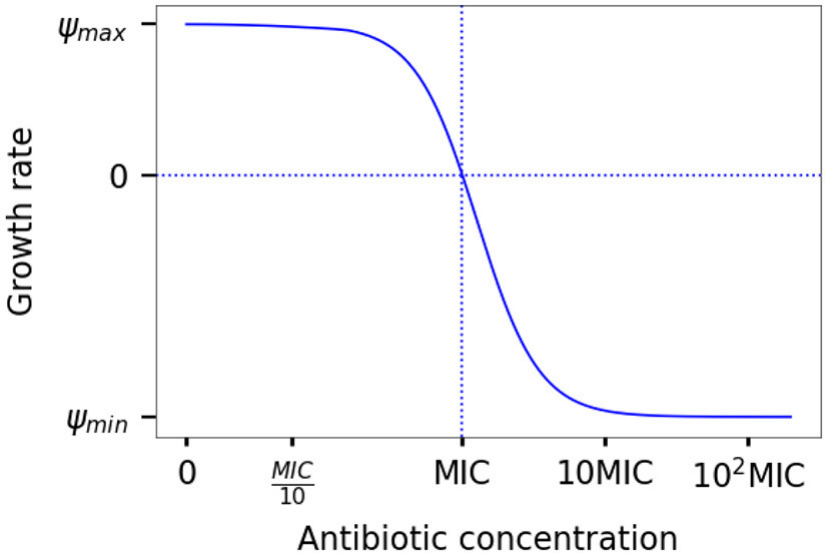
The pharmacodynamic function. The population growth rate Ψ depends on the antibiotic concentration *A* and pharmacodynamic properties, MIC, Ψ_*min*_, and Ψ_*max*_.

To quantitatively describe the interaction between antimicrobials and bacterial populations, we apply pharmacodynamic function. The pharmacodynamic function captures the net growth rate of a bacterial population Ψ as a function of the antibiotic concentration *A* (see Figure 1). The maximal bacterial growth rate defines the shape of the pharmacodynamic curve in the absence of antibiotic (Ψ_*max*_); the lowest net growth rate (Ψ_*min*_) that can be attained at very high antibiotic concentrations and is usually negative. The minimum inhibitory concentration (*MIC*) designates a concentration where the net growth rate crosses the horizontal axis and becomes negative. The steepness of the curve is determined by the Hill coefficient (κ) (Regoes et al., 2004):

(1) $$\begin{array}{l} \text{Ψ}={\text{Ψ}}_{max}-\frac{\left({\text{Ψ}}_{max}-{\text{Ψ}}_{min}\right){\left(\frac{A}{MIC}\right)}^{\kappa }}{{\left(\frac{A}{MIC}\right)}^{\kappa }-\frac{{\text{Ψ}}_{max}}{{\text{Ψ}}_{min}}}. \end{array}$$

Instead of Ψ_*min*_, the minimum duration can indirectly capture the maximum effect to kill 99% of the bacterial population (*MDK*_99_) (Balaban et al., 2019), or the antibiotic concentration at which the death rate is at half of its maximum (*EC*_50_).

Parameters of the pharmacodynamic function are determined by fitting the curve to bacterial growth rates at varying antimicrobials concentrations. The growth rates are estimated from time-kill data as the bacterial population size regression coefficients against time. Occasionally, especially in experimental studies where bacterial population sizes are estimated from optical density measurements, the pharmacodynamic function is shown to range between Ψ_*max*_ and 0, even for large antibiotic concentrations. Instead of MIC, a concentration that inhibits the growth to half of Ψ_*max*_ (*IC*_50_) is used. This is due to the nature of the optical density measurements that do not allow for observing negative growth rates. Even though both perspectives are similar at first glance, they are conflicting in their predictions of growth rates at high antibiotic concentrations. Therefore, they should not be used interchangeably, and special care should be taken when interpreting results.

Below, we explain individual modeling steps in detail, including biological explanation and justification.

### 2.1. Model Setup

#### 2.1.1. From Genotypes to Phenotypes

Bacteria are haploid, prokaryotic organisms. Therefore, we represent the bacterial genome by *k* biallelic loci. A genotype is represented by a bit-string (i.e., a vector of zeros and ones) of length *k*, where 0 represents a sensitive allele and 1 represents a mutated, resistant allele. There are 2^*k*^ possible genotypes.

In the presence of antibiotics, the mutants are killed at a slower rate. The MIC needed to stop the growth of the mutated bacterial population is higher. However, many mutations conferring antibiotic resistance are often *loss of function* mutations, disrupting the functioning of a particular molecule that would be a target or a transporter of the drug (e.g., binding site, resulting in a lower affinity to both the drug and the original molecule). Such disruption is typically considered maladaptive in the environment without the drug—as it comes at a fitness cost in the drug-free environment. This means that the replication rate of the resistant mutant is often slower in the drug-free environment, and the resistant mutations are either present at low frequencies or completely purged from the population. Note, however, that the trade-off for resistance mutation acquisition is still an open question (Melnyk and Kassen, 2011; Igler et al., 2021), and compensatory mutations might mitigate the fitness loss.

In our model, each resistant allele is associated with the benefit of increasing the fitness of the carrier in the presence of antibiotics and the cost of reducing fitness in the absence of antibiotics. Costs and benefits are given as vectors of length *k*, each element corresponding to the respective locus. Mutational effects of resistance-conferring mutation can be of various sizes: multiple mutations with small effects can combine (Wistrand-Yuen et al., 2018), likely in a multiplicative manner (Knopp and Andersson, 2018; Das et al., 2020). Therefore, when multiple loci are mutated in our model, their benefits and costs combine in a multiplicative way, and the total cost associated with genotype *i* is:

(2) $$\begin{array}{l} C_{i}=1-\underset{j}{\prod }\left(1-g_{ij}c_{j}\right) \end{array}$$

and the total benefit associated with genotype *i* is:

(3) $$\begin{array}{l} B_{i}={\text{g}}_{i}\cdot \text{b}=\underset{j}{\sum }g_{ij}b_{j}. \end{array}$$

##### 2.1.1.1. Effect of Lifestyle

In our model, we define two maximally distinct lifestyles: a planktonic lifestyle and biofilms. In the real world, planktonic populations consist of individual cells in liquid medium that are not constrained by space. Biofilms, on the other hand, are spatially highly organized, which leads to crowding, slower population growth, and potentially lower antibiotic concentrations within them. Rather than explicitly capturing the spatial differences between the lifestyles, we subsume them in the population- and pharmaco-dynamic parameters, specifically the replication rate of the bacteria and the MIC.

It has been proposed that biofilms owe their recalcitrance to their slow growth. Biofilm-inhabiting bacteria can grow slowly, either due to nutrient limitations (Roberts and Stewart, 2004) or due to the relatively high density of cells (Vrany et al., 1997). Other authors also argue that the fraction of persisters in the population is regulated by the cell density and thus is increased in biofilms (compared to planktonic cultures), similarly to the stationary state of planktonic cultures (Spoering and Lewis, 2001). In either case, the replication rate of the biofilm population is slower than that of the planktonic one, but the antimicrobial concentration needed to stop the biofilm growth is higher.

Here, we assume that the biofilm lifestyle confers additional, *non-heritable* cost *C*_*b*_ that decreases the fitness (replication rate) of the bacterial cells in the absence of antibiotics, and the total cost experienced by a mutated strain *i* living as biofilm is

(4) $$\begin{array}{l} C_{bi}=1-\left(1-C_{b}\right)\left(1-C_{i}\right). \end{array}$$

It also confers additional, *non-heritable* benefit *B*_*b*_ decreasing cells sensitivity to antibiotics. with the total benefit of a particular strain adopting a biofilm lifestyle given as:

(5) $$\begin{array}{l} B_{bi}=B_{i}+B_{b}. \end{array}$$

Note here that the biofilm lifestyle confers the same *absolute* benefit and *relative* cost to all strains, which is a simplification unlikely to hold in reality. It is yet to be determined how the protective effects of biofilm and resistant mutations combine.

If cells are released from the biofilm, they lose the biofilm-associated benefits and costs. In the planktonic lifestyle, benefits and costs are determined purely by the genotype, thus *B*_*pi*_ = *B*_*i*_ and *C*_*pi*_ = *C*_*i*_.

#### 2.1.2. From Phenotypes to Growth Rates

**Figure 2:**
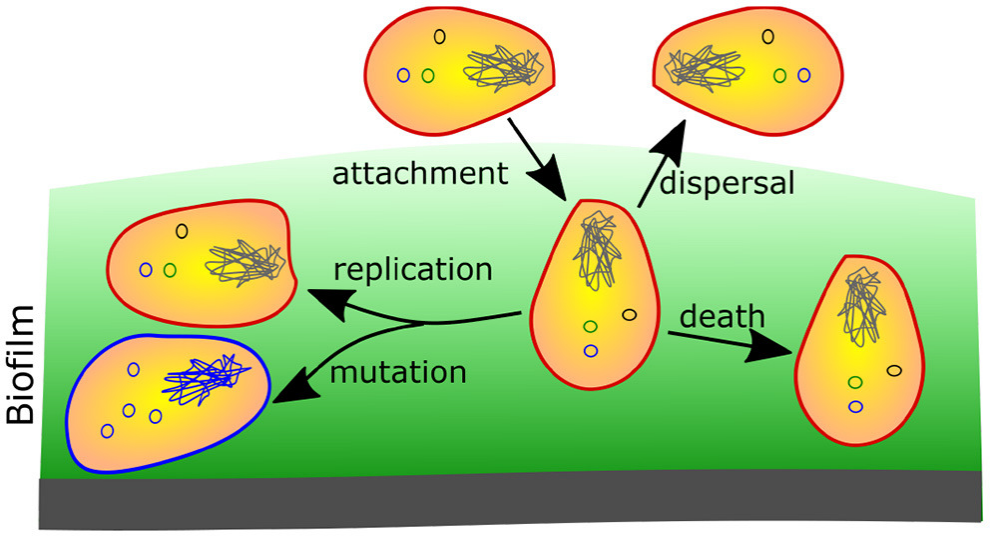
The main population dynamic processes in a biofilm that are used for the modeling. Bacterial cells die and replicate. During replication, they can mutate. Furthermore, planktonic cells can attach to biofilm, while biofilm cells can disperse and become free-living (plankton).

The following processes and their rates are modeled for each strain and lifestyle combination: bacterial replication, death, mutation, cell attachment, and dispersal (Figure 2). All of these processes occur concurrently and continuously. Therefore, the total change in the population size *N*_*i*_ of a particular biofilm inhabiting haplotype is given mainly by the rate of its replication and death (both natural and due to antibiotics, but is also increased by the attachment of the cells (with the same genotype) and decreased by their dispersal. Moreover, it is increased by “incoming” mutations that change other genotypes into the focal one and decreased by “outgoing” mutations that change cells of the focal genotype into a different one. While these contributions are usually negligible in large populations, they are essential for establishing new populations with mutated genotypes. For simplicity, we assume that antibiotics are completely *bactericidal*, and not *bacteriostatic*, meaning that they increase only the bacterial death rate and do not prevent bacterial reproduction.

##### 2.1.2.1. Replication

Both bacterial genotype and lifestyle influence their replication rate, and the joined cost of genetic and lifestyle manifest. The maximum replication rate of a particular mutated strain adopting planktonic lifestyle in the absence of antibiotics is given as:

(6) $$\begin{array}{l} {\text{Ψ}}_{0pi}={\text{Ψ}}_{s}\left(1-C_{pi}\right)={\text{Ψ}}_{s}\left(1-C_{i}\right). \end{array}$$

and if the same strain lives as a biofilm, it is

(7) $$\begin{array}{l} {\text{Ψ}}_{0bi}={\text{Ψ}}_{s}\left(1-C_{bi}\right)={\text{Ψ}}_{s}\left(1-C_{i}\right)\left(1-C_{b}\right). \end{array}$$

However, the population growth is limited by the nutrient availability, decreasing as the total population size approaches the carrying capacity of the environment *K*. This way, competition between various strains can be captured. Therefore, the replication of the i-th population is:

(8) $$\begin{array}{l} replication_{pi}=N_{pi}{\text{Ψ}}_{0pi}\left(1-N_{p}/K\right) \end{array}$$

for planktonic populations and

(9) $$\begin{array}{l} replication_{bi}=N_{bi}{\text{Ψ}}_{0bi}\left(1-N_{b}/K\right) \end{array}$$

for biofilm inhabiting strains, and *N*_*p*_ and *N*_*b*_ are the total population size across all possible strains adopting planktonic or biofilm lifestyles, respectively:

(10) $$\begin{array}{l} N_{p}=\sum N_{pi};N_{b}=\sum N_{bi}. \end{array}$$

Note that carrying capacity can be determined separately for biofilm and plankton, or both can be limited by the same carrying capacity, depending on the modeled scenario, in which case

(11) $$\begin{array}{l} N_{p}=N_{b}=\sum N_{pi}+\sum N_{bi}. \end{array}$$

##### 2.1.2.2. Mutations

Bacteria reproduce clonally. New variants are generated by mutation. Upon replication, each locus mutates with probability μ from 0 to 1. Given that many resistance mutations are *loss of function* mutations, backward mutations are rare enough to be excluded from the model; only mutations from non-resistant to resistant alleles occur (from 0 to 1). We further assume that double mutations occurring at the same time only happen at a negligible rate and do not consider them in this model. While many bacterial species are capable of horizontal gene transfer, it is at the moment omitted from this model. Thus, no recombination that would shuffle or create allele combinations is present.

Mutations rate from *j* to *i* determines, what fraction of strain *j* changes into strain *i*, subtracting it from *N*_*j*_ (“outgoing mutations”) and adding into *N*_*i*_ (“incoming mutations”). Mutated bacteria do not change their lifestyle. The mutations are dependent on replication—if this ceases (e.g., due to reaching the carrying capacity), mutation rate is also reduced:

(12) $$\begin{array}{l} mutations_{pi}=-\mu \left(k-\underset{j}{\sum }g_{ij}\right)replication_{pi}+\mu \sum replication^{*} \end{array}$$

in plankton and

(13) $$\begin{array}{l} mutations_{bi}=-\mu \left(k-\underset{j}{\sum }g_{ij}\right)replication_{bi}+\mu \sum replication^{*} \end{array}$$

in biofilm, where replication^*^ denotes replication of all populations that can mutate into genotype *i* within one step, meaning that new mutations are conditional on growth.

##### 2.1.2.3. Death

For simplicity, we assume that in the absence of antibiotics, all bacteria die at the rate of γ, independent of the lifestyle and genotype. However, both of these factors influence the sensitivity of the population to antibiotics—affecting the killing rate, modeled by the pharmacodynamic function. The final death rate in the presence of antibiotics is

(14) $$\begin{array}{l} death_{pi}=N_{pi}◦\left(\gamma +\frac{\left({\text{Ψ}}_{max\;pi}-{\text{Ψ}}_{min\;p}\right){\left(\frac{A}{MIC_{pi}}\right)}^{\kappa }}{{\left(\frac{A}{MIC_{pi}}\right)}^{\kappa }-\frac{{\text{Ψ}}_{min\;p}}{\text{Ψ}max\;pi}}\right) \end{array}$$

for plankton.

The final death rate in the presence of antibiotics is

(15) $$\begin{array}{l} death_{bi}=N_{bi}◦\left(\gamma +\frac{\left({\text{Ψ}}_{max\;bi}-{\text{Ψ}}_{min\;b}\right){\left(\frac{A}{MIC_{bi}}\right)}^{\kappa }}{{\left(\frac{A}{MIC_{bi}}\right)}^{\kappa }-\frac{{\text{Ψ}}_{min\;b}}{\text{Ψ}max\;bi}}\right) \end{array}$$

for biofilm, where

(16) $$\begin{array}{l} {\text{Ψ}}_{max\;pi}={\text{Ψ}}_{0pi}\left(1-N_{p}/K\right)-\gamma \end{array}$$

is the maximum net growth rate of the i-th planktonic population in the absence of antibiotics and

(17) $$\begin{array}{l} {\text{Ψ}}_{max\;bi}={\text{Ψ}}_{0bi}\left(1-N_{b}/K\right)-\gamma \end{array}$$

is the maximum net growth rate of the i-th biofilm inhabiting strain in the absence of antibiotics. In biofilm, minimal growth rate Ψ_*min b*_ is also altered by the biofilm lifestyle as Ψ_*min b*_ = Ψ_*min p*_(1 − *C*_*b*_).

##### 2.1.2.4. Attachment and Dispersal

Biofilm inhabiting bacteria disperse at rate ρ, independent of the strain. Similarly, free living bacteria can attach to the surface, or to the existing biofilm, at rate α. Dispersal and attachment represent exchange terms between plankton and biofilm lifestyle. Population sizes of bacterial populations are increased, and planktonic populations are decreased, at the rate

(18) $$\begin{array}{l} attachment_{i}=\alpha N_{pi} \end{array}$$

and population sizes of bacterial populations are decreased, and planktonic populations are increased, at the rate

(19) $$\begin{array}{l} dispersal_{i}=\rho N_{bi}. \end{array}$$

##### 2.1.2.5. Net Population Change

The net change of the population size of the i-th free living bacterial strain is:

(20) $$\begin{array}{l} \frac{dN_{pi}}{dt}=replication_{pi}+mutation_{pi}-death_{pi}-attachment_{i}+dispersal_{i} \end{array}$$

and the net change of the i-th biofilm inhabiting bacterial strain is:

(21) $$\begin{array}{l} \frac{dN_{bi}}{dt}=replication_{bi}+mutation_{bi}-death_{bi}+attachment_{i}-dispersal_{i}. \end{array}$$

Note that the actual number of the cells that attach or disperse in any step of our stochastic simulations is given by a random number drawn from a Poisson distribution with a mean given by the equations above. Therefore, single or multiple cells can found a biofilm, attach to or disperse from it.

#### 2.1.3. Environmental Parameters

The experiment, or the treatment, consists of any number of cycles that are defined by their length, starting antibiotic concentration, initial population size, carrying capacity of the environment, the drug degradation rate, and the dilution of the final population before it enters the next cycle. See Table 1 for details. Many different treatment regimes can be modeled by adjusting these parameters: from a single treatment through passages, commonly used in evolutionary experiments, to a chemostat environment.

**Table 1:** Symbols used in the model.

**Variable type**	**Symbol**	**Description**	**Variable type**
Genetics	0,1	Sensitive and resistant allele, respectively	
	*k*	Number of loci	Input
	***g*_*i*_**	Particular genotype/strain defined by a set of alleles	Calculated
	**b**	Vector of benefits of respective loci	Input
	**c**	Vector of costs of respective loci	Input
	μ	mutation rate	Input
Pharmacokinetic/ pharmacodynamics properties determined by drug-strain combination	Ψ_*s*_	Max replication rate of the sensitive strain living as plankton	Input
	Ψ_0*pi*_ (Ψ_0*bi*_)	Max replication rate of i-th resistant strain living as plankton (biofilm)	Calculated
	Ψ_*pi*_, (Ψ_*bi*_)	Replication rate of i-th resistant strain, when carrying capacity taken into account, in plankton (biofilm)	Calculated
	Ψ_*maxpi*_ (Ψ_*maxbi*_)	Max net population growth rate of i-th resistant strain in plankton (biofilm)	Calculated
	γ	Basal death rate	Input
	*MIC*_*s*_	MIC of a sensitive strain	Input
	*MIC*_*pi*_ (*MIC*_*bi*_)	MIC of i-th strain in plankton (biofilm)	Calculated
	κ	Hill coefficient	Input
	ρ	Drug degradation rate	Input
Population sizes	*N*_*pi*_	population size of a planktonic population with i-th genotype	Output
	*N*_*bi*_	Population size of a biofilm population with i-th genotype	Output
	*N*	Total population size	Output
Experiment	*K*	Carrying capacity of the environment	Input
	*A*	Antibiotic concentration	Input

### 2.2. Implementation

**Figure 3:**
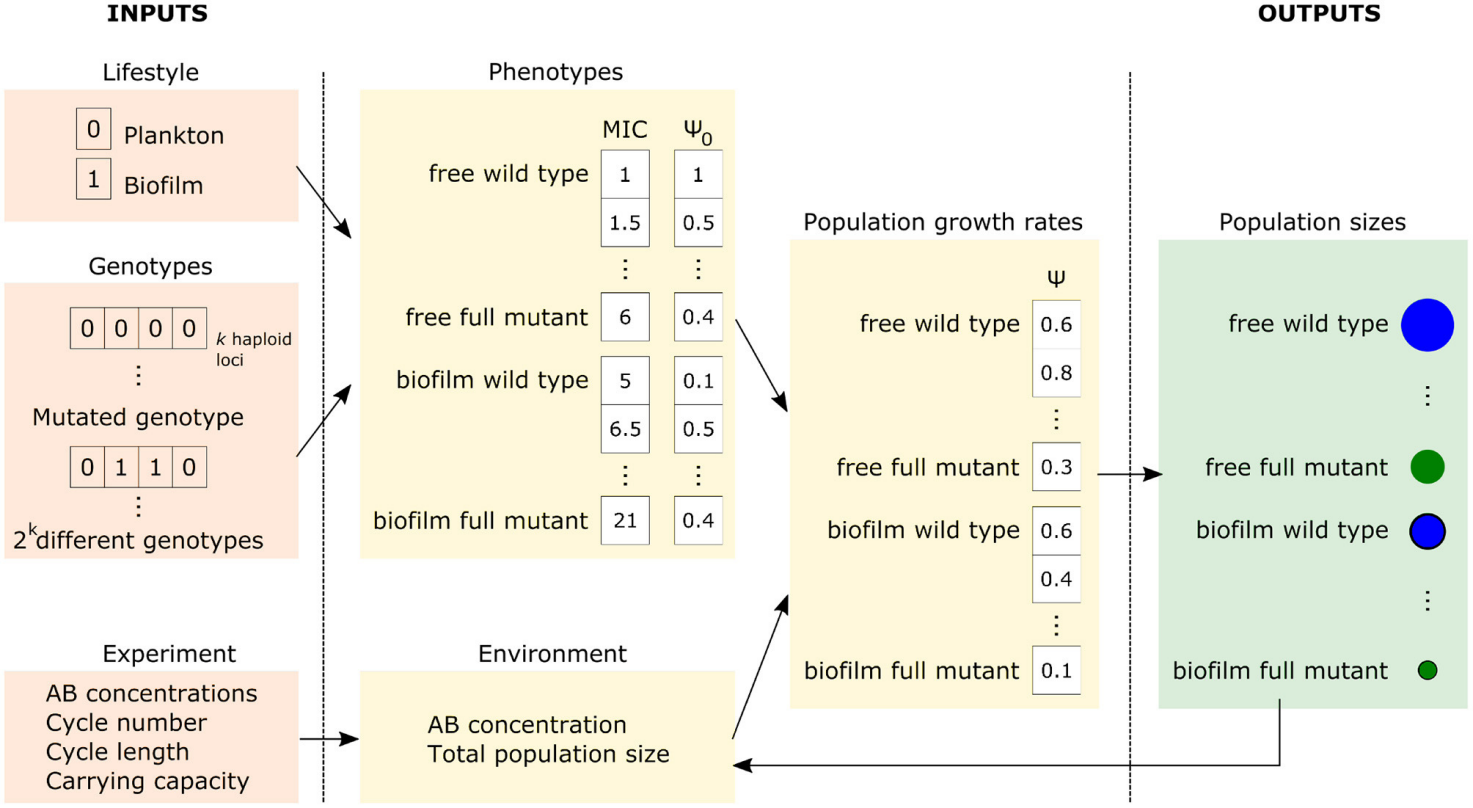
Simplified scheme of the model. Genotypes and lifestyles determine the phenotypes—the pharmacodynamic curve of each strain. The experiment setup defines the drug concentration (environment), which, together with the phenotype, determines each strain's growth rate and, subsequently, population size. Blue and green circles represent the sizes of the wild type and the mutant populations, respectively.

The model is implemented in Python3. Simulations are stochastic, using the Gillespie Tau Leaping algorithm. Inputs of the model are various parameters determining the genetics, the translation to phenotype and the details of the experiment (the treatment) (see Figure 3 and Table 1 for details). Simulations are typically initiated with a sensitive population of size *N*_*i* = 0_ = *N*_0_; however, the presence of resistant mutants in the initial population can also be modeled by specifying particular *N*_*i*_ in inputs.

The model's output is complete records of population sizes of all population types (strain and lifestyle combinations) in time. Therefore, it is possible to observe population dynamics (e.g., changes in population sizes, allele frequencies, or accumulation of the mutations), the timing of events (e.g., when a mutant appears or reaches some frequency), as well as calculate the emerging population properties (e.g., diversity) and observe their changes over time. Furthermore, stochastic simulations allow determining the probability distributions of the outcomes.

## 3. Simulated Scenarios

### 3.1. Effect of Drug Concentration on Resistance Evolution

Biofilm lifestyle, and especially phenotypic recalcitrance mechanisms inherent to it, are expected to influence resistance evolution, with non-intuitive outcomes depending on the concentration of antibiotics to which the populations are exposed. In Trubenová et al. (2022), we suggested that protection mediated by extracellular matrix shifts the *mutant selection window* (MSW), creating five distinct concentration ranges with different consequences for resistance evolution. The first set of simulations investigates these claims.

**Figure 4:**
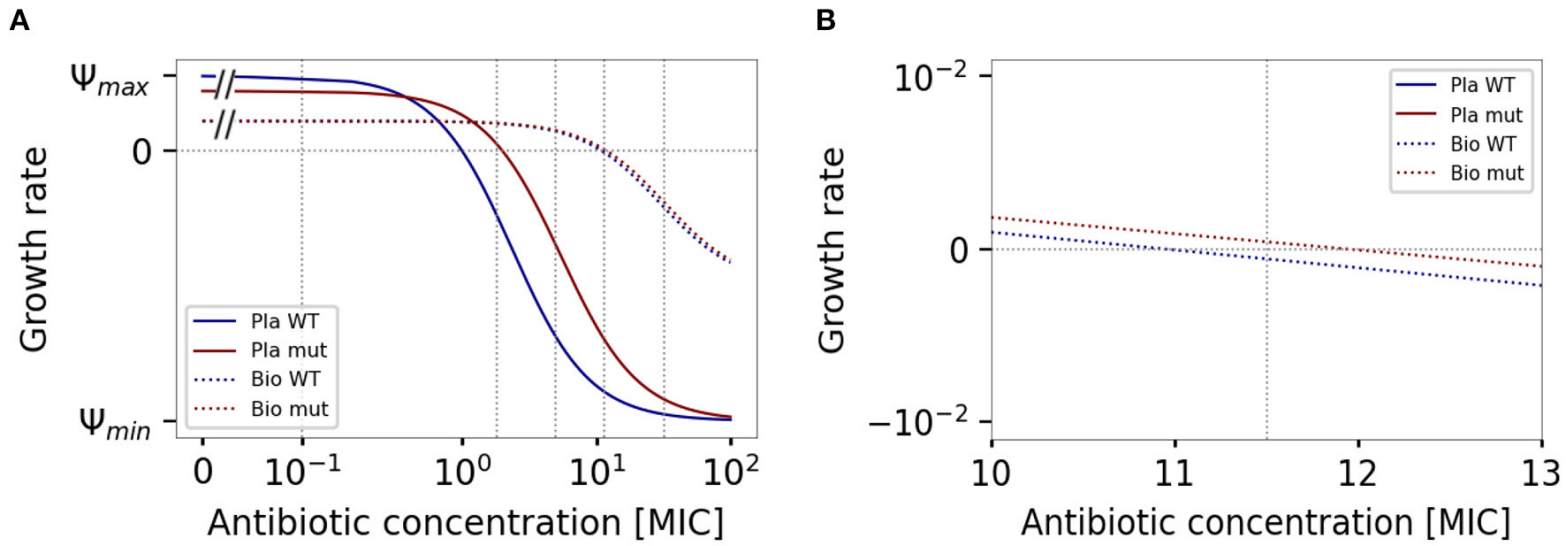
Pharmacodynamic curves of the wild type (blue) and the mutant (red) in both biofilm (dotted lines) and plankton (solid lines). Vertical dotted lines represent the initial drug concentrations used for the simulations. **(A)** Sampled concentration range on a log scale. **(B)** Detail of the MSW in biofilm, on a linear scale.

We first assume that resistance is encoded by a single locus (multi-locus scenarios are explored below), doubling MIC in planktonic cells but reducing the replication rate in the absence of antibiotics by 20%. Adoption of the biofilm lifestyle further reduces the replication rate in the absence of antibiotics Ψ_*max*_ as well as the minimum death rate Ψ_*min*_ by 50%; however, it increases the MIC by 10 MIC units in both the wild type and the mutant. The resulting four pharmacodynamic curves of the wild type and the mutant, living either planktonic or in a biofilm, divide the areas into five concentration ranges (see Figure 4). We chose one antibiotic concentration from each range: 0.1, 1.8, 5, 11.5, 32 MIC. Antibiotics degraded over time at the rate of 6 × 10^−4^ MIC units per minute. Simulations were initiated by an equal mix of wild type and resistant mutant (the starting population size of each was 5 × 10^5^). We run 50 stochastic simulations for each set of parameters. The growth of biofilm and planktonic populations were simulated independently for 24 h (or longer, if needed for illustration).

### 3.2. Polygenic Resistance Simulations

While the simulations described above consider a simple single locus scenario, here we take advantage of the polygenic nature of the model and investigate in detail the two most interesting, seemingly conflicting predictions—that biofilms can accelerate resistance evolution vs. the prediction that biofilms can slow down genotypic resistance evolution. Further, we disentangle the effects of two types of phenotypic mechanisms inherent to the biofilm lifestyle: (a) an extracellular matrix reducing drug penetration and (b) physiological alterations relying on a slow replication rate on resistance evolution. We simulate each of these mechanisms individually and in combination, comparing the results against the scenario with no phenotypic mechanisms involved (i.e., free-living bacteria):

*No phenotypic mechanisms:* Here, we assumed that no phenotypic mechanisms are in play; thus, there is no difference between planktonic and biofilm lifestyles, and we usually refer to this scenario as *Plankton*. Recalcitrance is only possible by acquiring true antibiotic resistance through genetic mutations. Note that here we assume that genetic resistance mechanisms in the biofilm are the same as in the planktonic cell, although these alone are not sufficient to explain the overall biofilm recalcitrance; the exact resistance mechanisms have been shown to contribute to resistance in biofilms as in planktonic cells but to a different degree (for example, drug efflux pumps were shown to be more recurrent in biofilms) (Dufour et al., 2010; Ciofu et al., 2017, 2022). Mutation rates may differ between lifestyles, as mutations themselves are likely different, with different costs and benefits (Santos-Lopez et al., 2019).

*EPS acting as a barrier:* We assumed that biofilm inhabiting cells are protected by reduced penetration mediated by EPS, increasing MIC by 10 MIC units. This was simulated by setting the biofilm benefit to 10 while keeping the biofilm cost at 0.

*Physiological alterations:* In the third scenario, we assumed that the recalcitrance is only caused by the altered physiology—low replication rates, resulting in the low death rates. This was simulated by setting biofilm benefit to 0 while setting biofilm cost to 0.9, thus reducing the maximal and minimal growth rates (Ψ_*max*_ and Ψ_*min*_, respectively) to 10%.

*Combined effects of physiology and EPS:* In the final scenario, we assumed that both mechanisms play a role—the replication (and subsequently death) rate is significantly reduced, while EPS confers additional protection. This was simulated by setting biofilm cost o 0.9 and biofilm benefit to 10 MIC.

In all scenarios, we assumed that genetic mechanisms conferring resistance were identical. Resistance was encoded by four identical and independent loci, each conferring a benefit of 2 (doubling MIC) and a cost of 0.1 (10% reduction in fitness in the absence of antibiotics). Parameters used were biologically realistic, informed by experiments performed in biofilms with *Staphylococcus aureus* (e.g., doubling rates, mutation rates, dilution factors, antibiotic concentrations, see Supplementary Table 1 for details). The drug was degraded at the rate of 6 × 10^−4^. The initial population size consisted of only sensitive wild type with $N_{0}=10^{6}$. The carrying capacity of the environment was set to 10^9^. For each parameter set, we run 100 stochastic simulations. Note that only one lifestyle was simulated in each simulation (no co-existence of plankton and biofilm). All four scenarios were used to investigate the population dynamics in two types of simulated treatments:

#### 3.2.1. Repeated Treatment

To mimic a real-life situation common in clinical practice (Michiels et al., 2016)—a treatment of bacterial infection by a particular dose of antibiotics in regular intervals (Duan et al., 2021)—we simulated a 7-day treatment in which populations were treated with antibiotics at concentration *A* every 24 h, and the population sizes were *not* externally modified (diluted). We used five different concentrations: 5, 10, 15, and 20 MIC.

In these simulations, we investigated the survival of the populations—if and when the bacterial populations became extinct, and if they survived the whole treatment, what was the final genetic composition of the evolved population—i.e., whether resistance evolved and to what degree.

#### 3.2.2. Passage Experiments

Furthermore, we simulated a typical passage experiment with exponentially increasing concentration of antibiotics (Santos-Lopez et al., 2019; Scribner et al., 2020; Duan et al., 2021). The initial concentration of antibiotics was 0; then 0.5 MIC, and then it doubled every 24 h until 64 MIC on day 9. The drug was degraded over time. If the population grew over 24 h, it was diluted to the original size. If not, it was left intact, but the antibiotic concentration was increased.

### 3.3. The Effect of Interaction Between Biofilm and Plankton

In the final set of simulations, we investigated the effects of the most natural scenario—the co-existence of biofilm and planktonic populations and their interaction via continuous dispersal and attachment. Two populations were simulated in parallel, coupled with equal attachment and dispersal rates. We used five different rates: 0, 0.0001, 0.001, 0.01, 0.1 [1/min], and performed 20 stochastic simulations of the passage experiment for each rate.

## 4. Results

**Figure 5:**
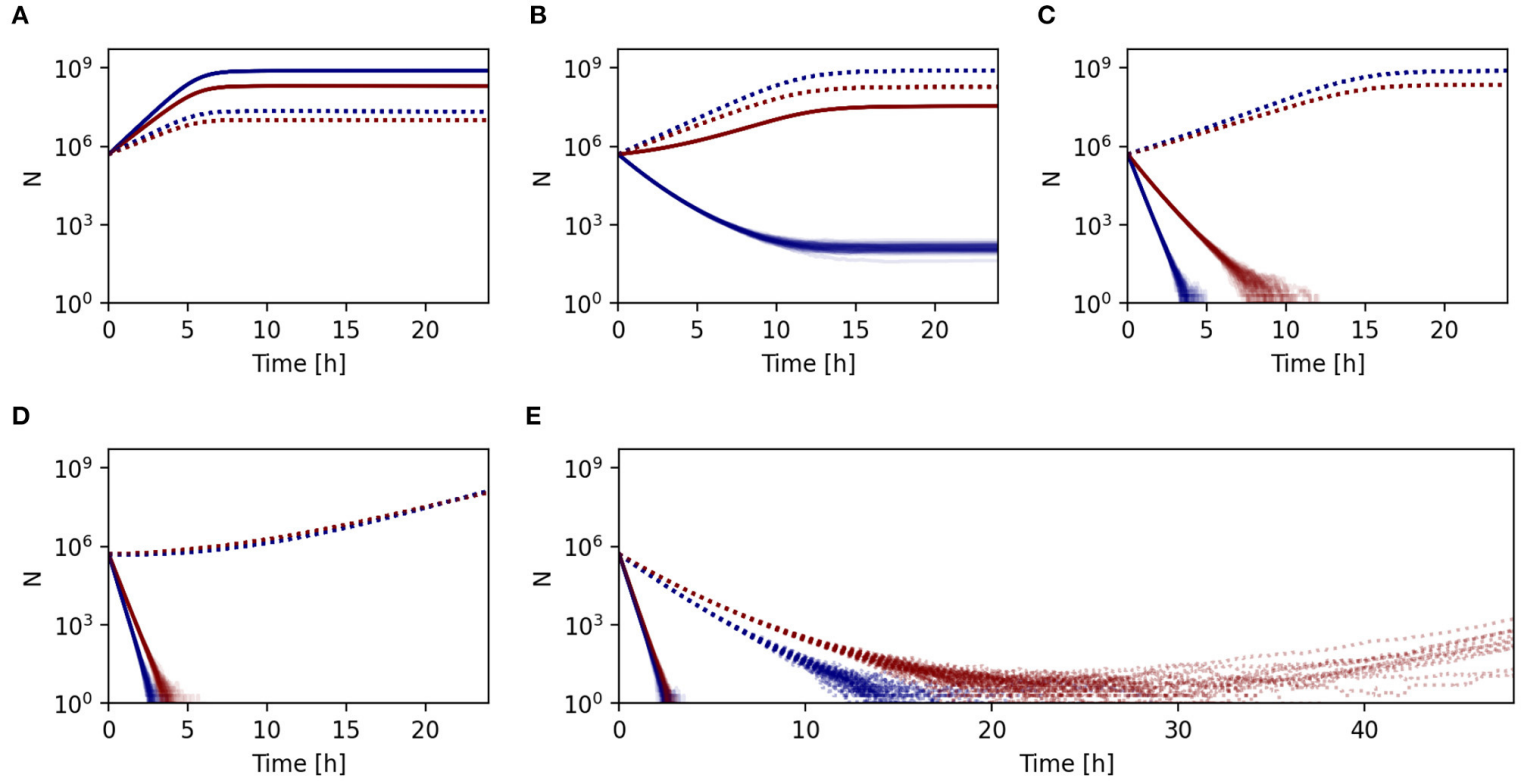
Whether resistance evolved in either planktonic (solid lines) or biofilm (dashed lines) population depends on concentration of antibiotics the populations experience. Blue lines represent wild type, red lines represent mutant populations. Initial concentrations [MIC]: **(A)**: 0.1, **(B)**: 1.8, **(C)**: 5, **(D)**: 11.5, **(E)**: 32.

The population dynamics of the sensitive wild type and resistant mutants differ in various environments. At the lowest concentration, the susceptible wild type has higher fitness than mutants, regardless of the adopted lifestyle (Figure 5A). At higher concentrations, however, within the mutant selection window the growth rate of the mutant is positive and higher than the growth rate of the wild type (Drlica, 2003; Drlica and Zhao, 2007; Yu et al., 2018) in planktonic cultures. While the sensitive, free-living wild type may go extinct, free-living mutants survive, leading to antibiotic resistance evolution and spread. This is, however, not the case in the biofilm, where the mutation is still detrimental, and sensitive biofilm-inhabiting bacteria grow faster than the mutants, inhibiting the evolution of resistance (Figure 5B). Depending on the phenotypic protection mediated by the extracellular matrix, resistance evolution may be prevented even in the concentration range where the free-living strains are not able to survive and are killed by the antibiotics (Figure 5C).

In the biofilm mutant selection window, protection mediated by extracellular matrix no longer suffices to protect the biofilm inhabiting sensitive wild type, while the resistant mutant still survives. This is the concentration range facilitating the evolution of antibiotic resistance. Note, however, that while a single treatment with antibiotics in this concentration range may suffice to clear the planktonic cells, it might not remove sensitive biofilm bacteria if the antibiotics are degraded fast enough, as can be seen in our simulations (Figure 5D). In our simulated example, the pharmacokinetic and pharmacodynamic properties of the drug and strain combination allowed even the sensitive biofilm-inhabiting bacteria to survive long enough until the drug was degraded and subsequently restarted its growth.

Finally, even if the concentration of antibiotics is higher than the MIC of the mutants and their population is likely to decline, if the death rate is sufficiently reduced by the presence of persisters or by the low metabolism, resistant bacteria might be able to survive long enough to restart the growth after sufficient drug degradation. In most of our simulations, the combined effect of phenotypic and genetic mechanisms (mutations) led to the survival of the mutated biofilm cells long enough until the antibiotics got sufficiently degraded. While population sizes initially dropped in all simulations, some were able to recover (Figure 5E). Note, however, that at a high enough concentration, even mutated biofilm inhabiting cells would likely not be able to survive long enough until the drug is sufficiently degraded.

The dose of antibiotics used for the treatment of bacterial infections is often around 5 to 10× MIC. This means that biofilms are likely facilitating the development of resistance in clinical practice. High antibiotic concentrations for a prolonged period of time may be needed for the treatment of biofilm infections (Høiby et al., 2015).

Below, we focus in detail on the two most interesting and seemingly conflicting effects of recalcitrance mechanisms inherent to the biofilm lifestyle on resistance evolution: depending on the scenarios considered, resistance evolution can either be suppressed or facilitated. We disentangle the effects of phenotypic protection mediated by the extracellular matrix and physiological alterations in the context of resistant mutants and show how they influence resistance evolution individually and in combination. Furthermore, we investigate the effects of other system properties—e.g., treatment regime or experimental setup.

### 4.1. Biofilm Lifestyle Suppresses Resistance Evolution at Low Antibiotic Concentrations

**Figure 6:**
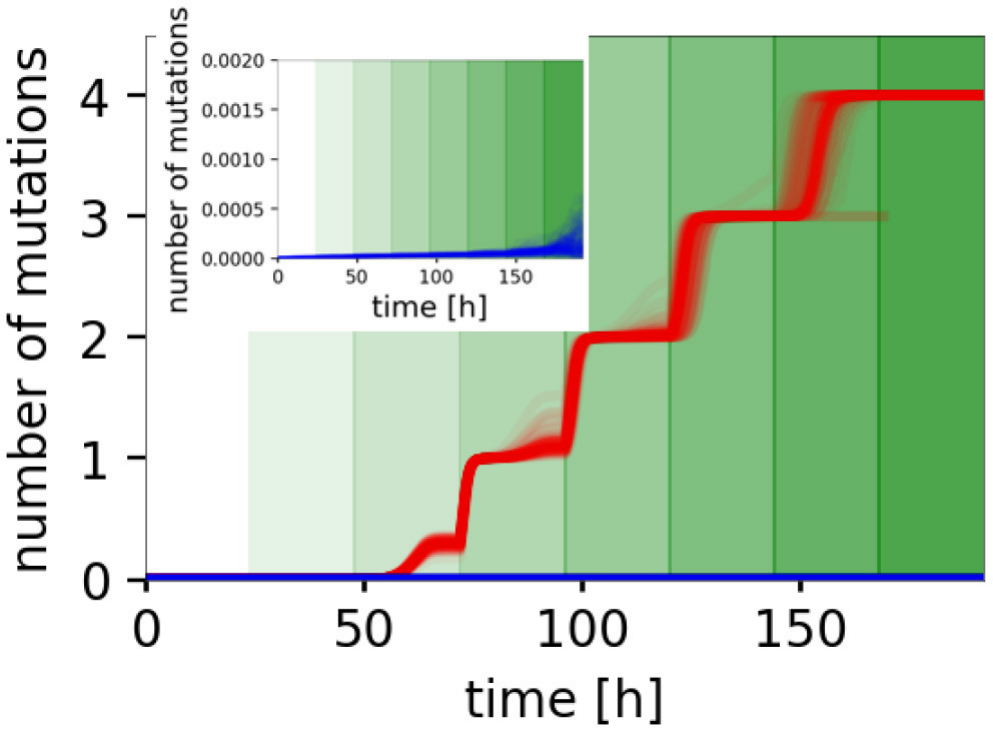
Accumulation of mutations. The average number of mutations per cell in planktonic culture (red) and biofilm (blue) when both phenotypic mechanisms are considered. See Supplementary Material for individual effects. The switch in the shades of green represents times when the antibiotic concentration is doubled. Showing 100 stochastic simulations for each scenario.

When planktonic populations are exposed to sufficiently high concentrations of antibiotics, only those that acquire beneficial mutations will survive. Figure 6 illustrates the accumulation of mutations in plankton and biofilm during a passage experiment. The simulations show that the average number of mutations per bacterial cell increased quickly in planktonic culture, where a new mutation was necessary for survival after each concentration increase. By contrast, the biofilm lifestyle prevented mutation accumulation when the combined effects of reduced penetration and physiological alterations were considered.

Interestingly, in simulations, where only protection mediated by EPS and reduced penetration was considered, decreased selection pressure and the need for mutations led to very low, almost no mutation accumulation (Supplementary Figures 1, 2). While these mutations appeared, they remained at low frequency. Once the protection mediated by EPS did not suffice to protect the biofilm-inhabiting bacteria, the biofilm population did not manage to evolve resistance fast enough and died out, even before the planktonic populations (see Supplementary Figures 1A,B).

When only physiological alterations were considered, resistance evolution was hampered by slow replication and thus a low probability for mutations to arise. While some mutations appeared and the mean number of mutations increased, the biofilm died even sooner (at low concentrations) than when the reduced penetration only was considered (Supplementary Figure 1C). Therefore, we see both the lack of opportunity (due to a lower mutation rate) and the absence of selection pressure that slows down the resistance evolution in biofilms, especially at low concentrations.

However, the combination of both mechanisms allowed the biofilm to survive the treatment—EPS mediated protection kept the population size high enough for a long time. Then the slow death rate enabled its survival until the end of the experiment, even though the population size was slowly declining (Supplementary Figure 1D). Note, however, that the chosen parameters heavily influence the dynamics. For instance, imposing a lower cost of biofilm lifestyle (50% reduction in replication and death rate—instead of the default assumption of 90%) led to different dynamics. In this case, a low reduction of replication rate allowed mutations to accumulate quickly, even in the biofilm when only physiological alterations were considered. This, however, was counterbalanced when combined with the reduced selection pressure and led to biofilm extinction (Supplementary Figures 3, 4).

### 4.2. Biofilm Lifestyle Enables the Resistance Evolution Under High Concentrations

In contrast to the situation of low antibiotic concentrations discussed above, a biofilm lifestyle is expected to increase the survival of bacterial populations under high antibiotic concentrations and also facilitate the evolution of genetic resistance. This effect becomes most obvious when bacterial populations are treated repeatedly with a particular concentration of antibiotics.

Simulations of the repeated treatment show that drug doses resulting in high enough concentrations prevented the survival of the planktonic cultures and the evolution of resistance in them (see Supplementary Figure 6 for an example of population dynamics), even though the planktonic population was able to adapt to such concentrations if these increased gradually (Supplementary Figure 1). Biofilm-inhabiting bacterial populations not only survived the treatment but evolved resistance. Higher antibiotic concentrations are needed to kill biofilms sufficiently quickly before resistance evolves.

**Figure 7:**
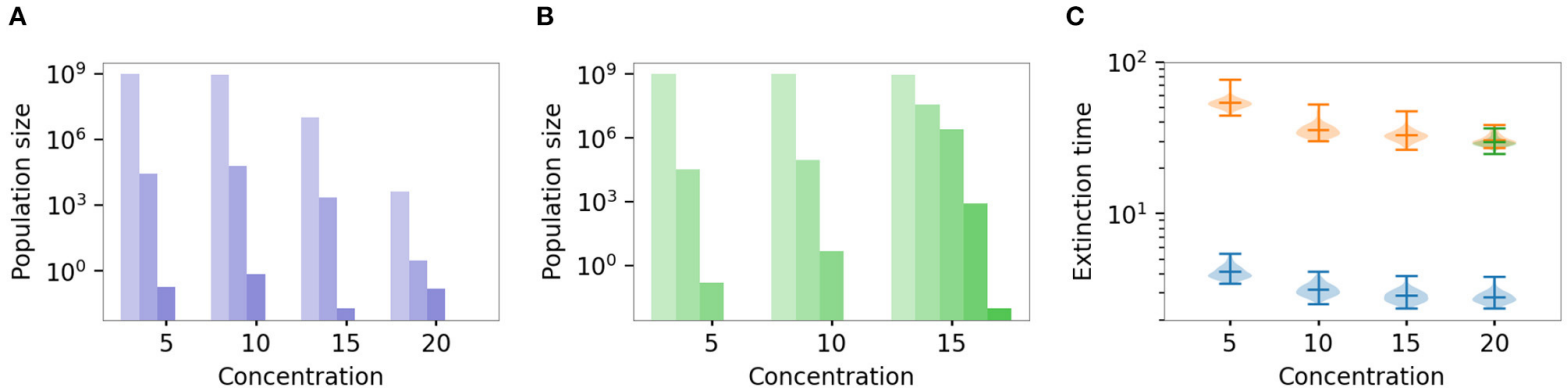
Effect of drug concentration on genetic composition and survival time. Different colors represent different scenarios: plankton, genetic mechanisms only (blue); biofilm relying on physiological alterations only (red); biofilm relying on EPS protection only (green), biofilm protected by the combination of both mechanisms (purple). Panel **(C)** is a complement of panels **(A,B)**—if the populations survived, we show its genetic composition **(A,B)**, if not, we show distribution of extinction times **(C)**. **(A,B)** Genetic composition of the final populations that survived 7-day treatment. Mean sizes across 100 simulations. Different shades of the same color represent subpopulations with different numbers of mutations: lightest for wild type and darkest for the full (quadruple) mutant. **(A)** Both extracellular matrix protection and physiological alterations are considered in combination. **(B)** Only extracellular matrix protection is considered. Populations did not survive treatment with concentration *A* = 20 MIC, and the distribution of extinction times is shown in **(C)**. **(C)** Distribution of extinction times for treatment with various concentrations. Note the log scale of the y-axes. For the distributions of extinction times of all simulations on a linear scale, see Supplementary Figure 5.

If the populations survived the treatment, we investigated the genetic composition of the final surviving population (Figures 7A,B). If they died during the treatment, we looked at the distribution of the extinction times (Figure 7C). Figure 7A shows the genetic composition of the biofilm population that survived the 7-day treatment for increasing concentrations when both phenotypic mechanisms, extracellular matrix protection, and physiological alterations were considered. The populations survived in all simulations. Figure 7B shows the genetic composition of the surviving biofilm population when only extracellular matrix protection was taken into account. In this case, the biofilm population was not able to survive the treatment with the highest concentration. When only physiological alterations were considered, biofilms did not survive the 7-day treatment at any concentration. Similarly, planktonic populations did not survive any treatment in any simulation. Figure 7C shows the distribution of the extinction times, as well as extinction times of the biofilm population at the highest concentration treatment when extracellular matrix protection was considered.

### 4.3. Continuous Interaction Between Biofilm and Plankton Increases the Probability of Resistance Evolution

While in our previous simulations, we simulated only a single population (either plankton or biofilm), here we focus on a more natural scenario—the co-existence of biofilm and planktonic populations and their interaction via continuous dispersal and attachment. Our results show that neither the complete absence of exchange between planktonic and biofilm compartments nor very high rates of attachment and dispersal are beneficial for biofilm survival and resistance evolution. Instead, intermediate rates seem to lead to prolonged survival and the fastest resistance evolution.

As discussed above, when biofilm and planktonic populations are isolated, planktonic populations evolve resistance but go extinct when concentrations become too high for mutants to survive. In contrast, biofilm-inhabiting bacteria are shielded from selection. Mutants appear rarely and do not increase in frequency. When the protection mediated by the extracellular matrix no longer suffices to protect the population, biofilm population may die out before sufficient mutations can rescue it.

**Figure 8:**
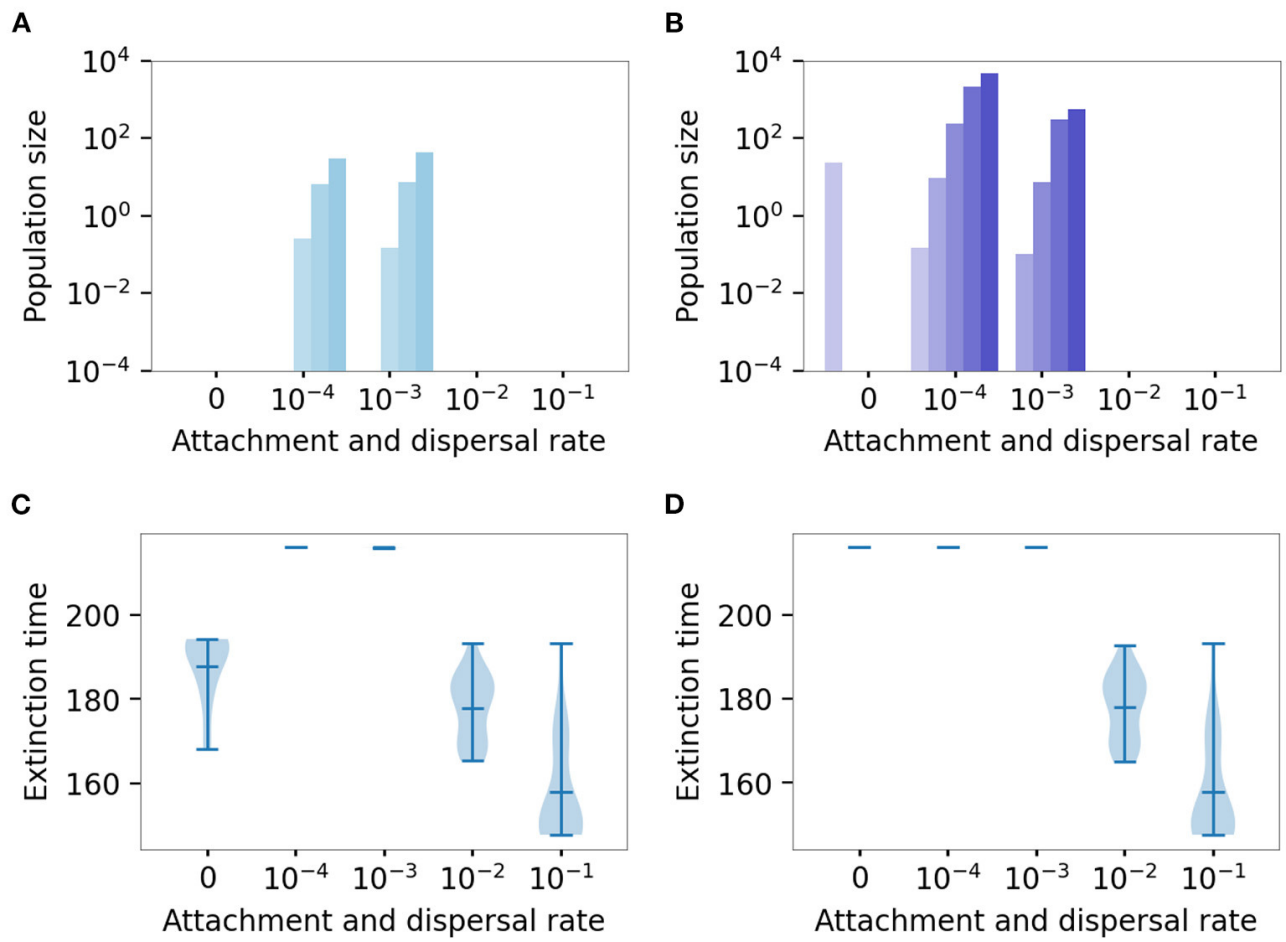
Effect of continuous attachment and dispersal on genetic composition and survival time. Genetic composition of the final populations that survived the passage experiment, in plankton **(A)** and biofilm **(B)**, where both physical, and physiological recalcitrance mechanisms were considered. The bars are colored blue (plankton) and purple (biofilm) in accordance with Figure 7. The degree of shading of the bars denotes subpopulations with a different number of mutations: lightest for wild type, darkest for the full (quadruple) mutant. Mean sizes across 20 simulations. Distribution of extinction times of plankton **(C)** and biofilm **(D)**.

However, when plankton and biofilm populations are connected via dispersal and attachment, biofilm populations can gain the mutations and then survive in the highest concentration. This interaction combines the advantages of both lifestyles—while the mutants appear and increase in frequency quickly in planktonic populations, evolving high levels of resistance, switching to the biofilm lifestyle will extend their survival beyond that of their planktonic counterparts. As before, when the populations survived the treatment, we investigated the genetic composition of the final, surviving population (Figures 8A,B), and if not, we plotted the distribution of the extinction times (Figures 8C,D).

Figure 8 shows the effect of low dispersal and attachment rates on the population composition of both planktonic (Figure 8A) and biofilm population (Figure 8B). At inter-medium rates (10^−4^ and 10^−3^), even the full mutant is present in both planktonic populations and the biofilm, while lower and higher rates led to the extinction of the population (see Supplementary Figure 7). An exception is a biofilm in isolation (attachment and dispersal rate is 0) that survived the treatment due to a slow death rate (see dynamics and discussion of the passage experiment in the first result section).

Thus, systems in which there is sufficient exchange between planktonic and biofilm compartments are the most critical with regard to resistance evolution and treatment failure.

#### 4.3.1. Following the Path of Evolution

**Figure 9:**
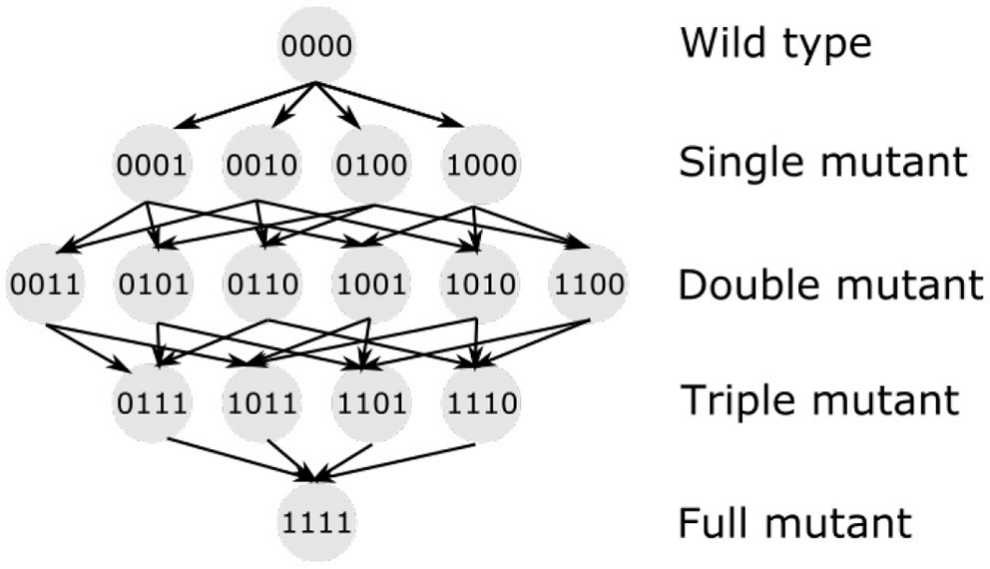
Evolutionary paths from a wild type to a full mutant. When four loci are considered, a wild type can mutate into four different single mutants, which, in turn, can mutate into three double mutants each, altogether six double mutants. Then the opportunity to mutate is reduced, with only four triple mutants available. Each has only one possible mutation left, converging into a single strain of full mutant. Circles with numbers—different genotypes (0—sensitive, wild-type, 1—resistant, mutated allele). Arrows show the direction of mutations present in the model.

**Figure 10:**
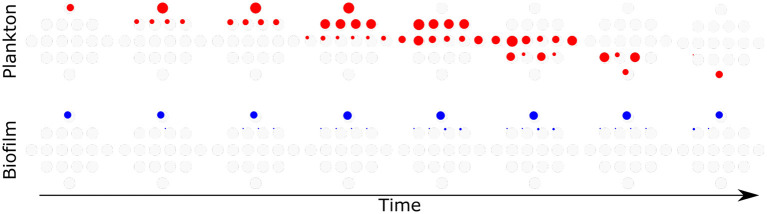
No attachment and dispersal. Red—plankton, Blue—biofilm. From left to right—time, population composition is analyzed every 24 h. The diameter of the colored circle is proportional to the log 10 size of the population. Light gray circles are placeholders for various genotypes, with a diameter corresponding to the carrying capacity of the environment.

The path from the wild type to the full mutant may lead through many different paths, shown in Figure 9. As mentioned above, it is likely that the mutations ensuring biofilm survival at a high antibiotic concentration (above biofilm MIC) originate in planktonic cells rather than biofilms. The model follows all possible genotypes separately, allowing discrimination between single mutants with different mutations. We looked in detail into two simulations, observing which mutants were present in biofilm and in plankton at which time. Figure 10 shows the genetic composition of plankton and biofilm populations in the absence of attachment and dispersal. Each diamond shape represents all possible genotypes (as shown in Figure 9) at different times, from day 1 to day 8, at the beginning of the day. In plankton, composition changes from the wild type to the full mutant, while only single mutants are present in biofilm. There is no similarity in patterns between the population dynamics in these two populations.

**Figure 11:**
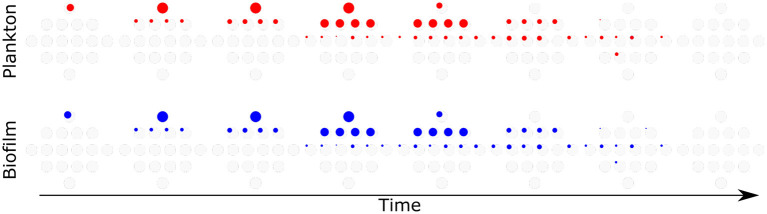
Attachment and dispersal 0.1. Red—plankton, Blue—biofilm. From left to right—time, population composition is analyzed every 24 h. The diameter of the colored circle is proportional to the log 10 size of the population. Light gray circles are placeholders for various genotypes, with a diameter corresponding to the carrying capacity of the environment.

On the other hand, Figure 11 shows a simulation with a high attachment and dispersal rate of 0.1. The genetic composition patterns across time of both plankton and biofilm are very similar. Note that the same strains are absent from both populations at the same time (e.g., 1010 mutant on day 7).

## 5. Conclusion

Many mechanisms contribute to the recalcitrance of biofilms against antimicrobials. Non-genetic mechanisms can be crucial for driving resistance evolution, as they alter the expression of the genotype and interfere with evolutionary processes. However, the individual and combined effects of these mechanisms are difficult to study experimentally. Given the multiple, possibly conflicting effects of biofilm lifestyle on the evolution of antibiotic resistance, population genetics models are a useful tool for clarifying the population genetic effects of each mechanism on their own and in combination. For instance, Roberts and Stewart (2004) modeled antibiotic tolerance (recalcitrance) by accounting for nutrient limitation. Adaptive responses to antimicrobial agents in biofilms were modeled by Szomolay et al. (2005). However, more comprehensive models accounting for the polygenic nature of antibiotic resistance and the complex phenotypic aspects of biofilm recalcitrance are still largely missing.

The model proposed here offers new quantitative insights into the evolution of resistance, population dynamics, and consequences of phenotypic recalcitrance mechanisms on antibiotic resistance evolution. By exploring the combinations of mechanisms that contribute to biofilm recalcitrance using computer simulations, we add quantitative support for two previous verbal predictions (Trubenová et al., 2022). First, we hypothesized that biofilms could slow down resistance evolution. In our simulations, this holds true under low antibiotic concentrations that are sufficient to select for antibiotic resistance in planktonic cultures (Gullberg et al., 2011; Santos-Lopez et al., 2019). Second, we showed that biofilms could promote resistance evolution under drug concentrations that are high enough to suppress most resistant planktonic mutants. Finally, when we allowed for a continuous exchange between planktonic cells and biofilms, the evolution of resistance was further accelerated.

Our simulations hence show that evolutionary outcomes of populations experiencing selection pressure from the presence of antibiotics are expected to depend heavily on many parameters, most notably the concentrations of antibiotics and the treatment regimen. The timing of treatments and frequency of dosing will influence the population dynamics of sensitive and resistant strains and determine the probability of resistance evolution.

Our modeling study makes many assumptions, in part due to uncertainty about the parameters that characterize the processes involved and, in another part, for conceptual and computational simplicity. Below, we briefly mention and discuss these assumptions and the limitations to which they lead and briefly mention how we could approach a more comprehensive quantitative description of the pharmacodynamics and population genetics of bacterial populations that grow as biofilms.

The values of parameters used in the simulations were taken from real-world scenarios whenever possible (see Supplementary Table 1). For instance, the degradation rate used in our simulations (rate 6 × 10^−4^ MIC/min, which translates into the drug half time of approximately 19 h) is consistent with *in vitro* measures (Lallemand et al., 2016): in soil it has been shown that the typical halftime of antibiotics is 2 − 80 days, depending on the soil composition and other conditions (Pan and Chu, 2016). In our simulations, the effect of the degradation rate is therefore only visible in the first set of illustrative simulations, while in other simulations (repeated treatment and evolutionary experiment), it does not have any noticeable effect.

However, in living organisms treated for infections, drugs are actively degraded and excreted, and drug concentrations decrease faster. Therefore, a significantly higher degradation rate would be more suitable for designing and analyzing treatment strategies.

When the parameter values were not known, and not even their magnitude could be estimated (e.g., dispersal and attachment rates), we investigated a range of these values across several orders of magnitude. While we could gain qualitative insights into the impact of exchange between biofilms and planktonic populations on resistance evolution despite these uncertainties in the dispersal and attachment rates, better estimates of these rates would allow us to gain quantitative insights into the impact of exchange.

In most of our simulations, we assumed a starting population without pre-existing resistant strains. This allowed us to investigate the emergence and the subsequent selection of resistant strains. Had we assumed pre-existing resistance in the simulations, we would have been able to obtain insights into the selection phase only. Furthermore, starting with a population consisting purely of wild-type strains allowed us to identify whether the mutants are more likely to appear in plankton or biofilm, a topic we discuss in Section 4.3. To investigate the effects of various treatment strategies, initiating the model with a bacterial population that already contains resistant mutants would be an important addition to the analysis presented here because, in many infections, resistant mutants are either co-transmitted with the wild type or arise *de-novo* before treatment in the infected host. Our modeling approach was designed to investigate the effects of recalcitrance mechanisms known in biofilms on the evolution of resistance without explicitly describing the intricate spatial characteristics of biofilms. Instead, we based our investigations on the population- and pharmaco-dynamic differences between the two lifestyles, some of which are direct consequences of the spatial aspects. As a result, in our simulation, planktonic bacterial populations and biofilms differ only in terms of their growth rates and pharmacodynamic parameters.

By defining homogeneous planktonic populations and biofilms this way, we ignore the physicochemical and physiological heterogeneity of biofilms and the heterogeneous physiological states between these two extreme lifestyles (e.g., persister cells in the plankton). Formally, these aspects could be incorporated into the modeling framework, stopping short of a full-blown spatial simulation of bacterial populations by introducing multiple subpopulations. In particular, multiple populations of the same strain but with different “biofilm related” properties could be added, simulating multiple layers of a biofilm. Similarly, heterogeneity could be introduced into planktonic populations as was done in, for example, (Balaban, 2004; Wiuff et al., 2005; Levin-Reisman et al., 2017; Rodriguez-Rojas et al., 2021). A promising avenue of future research will be to investigate if the bacterial lifestyles and their intrinsic heterogeneities facilitate or hinder resistance evolution.

## Data Availability Statement

The original contributions presented in the study are included in the article/Supplementary Material, further inquiries can be directed to the corresponding author/s. The code used to generate the results in this article is available at https://github.com/Trubenova/FrontiersBiofilmRecalcitrance.

## Author Contributions

BT implemented the model, performed the simulations, and wrote the first draft of the manuscript. All authors contributed to the conception and design of the study, analyzed the simulation results, developed the model, wrote sections of the manuscript, and contributed to manuscript revision, read, and approved the submitted version.

## Funding

This work was supported by a grant from the Volkswagen Foundation (Grant Numbers 96517 and 96695). Open access funding provided by ETH Zürich.

## Conflict of Interest

The authors declare that the research was conducted in the absence of any commercial or financial relationships that could be construed as a potential conflict of interest.

## Publisher's Note

All claims expressed in this article are solely those of the authors and do not necessarily represent those of their affiliated organizations, or those of the publisher, the editors and the reviewers. Any product that may be evaluated in this article, or claim that may be made by its manufacturer, is not guaranteed or endorsed by the publisher.
