# Supplementary material for: Modeling Polygenic Antibiotic Resistance Evolution in Biofilms
Source: Front Microbiol. 2022 Jul 7;13:916035. doi: 10.3389/fmicb.2022.916035 (PMC9301000; doi:10.3389/fmicb.2022.916035)
Supplement: Supplementary file 1 [file Data_Sheet_1.PDF]

Supplementary Material

1 SUPPLEMENTARY DATA

1.1 Values of the parameters used in the model

Table S1. Parameters used in the model

Symbol	Description	Value used	Unit	Source
Ψ_s	max growth rate of the sensitive strain	0.0231	[1/min]	Corresponds to the doubling time of 30 min, which is a reasonable estimate (Miller et al., 2021)
$\Psi_{min\ i}$	minimal growth rate	-0.0833	[1/min]	Corresponding to -5 per hour (Igler et al., 2021)
γ	basal death rate	7×10^{-5}	[1/min]	
N_0	starting population size	10^6		typically used by the authors in passage experiments
K	carrying capacity of the environment	10^9		observed by the authors in passage experiments
κ	Hill coefficient	1.5		(Igler et al., 2021)
d	antibiotic degradation rate	6×10^{-4}	[MIC/min]	(Pan and Chu, 2016; Lallemand et al., 2016)
μ	mutation rate	3×10^{-6}	[1/division]	(Igler et al., 2021)

1.2 Supplementary figures

1.2.1 Slowing down resistance evolution

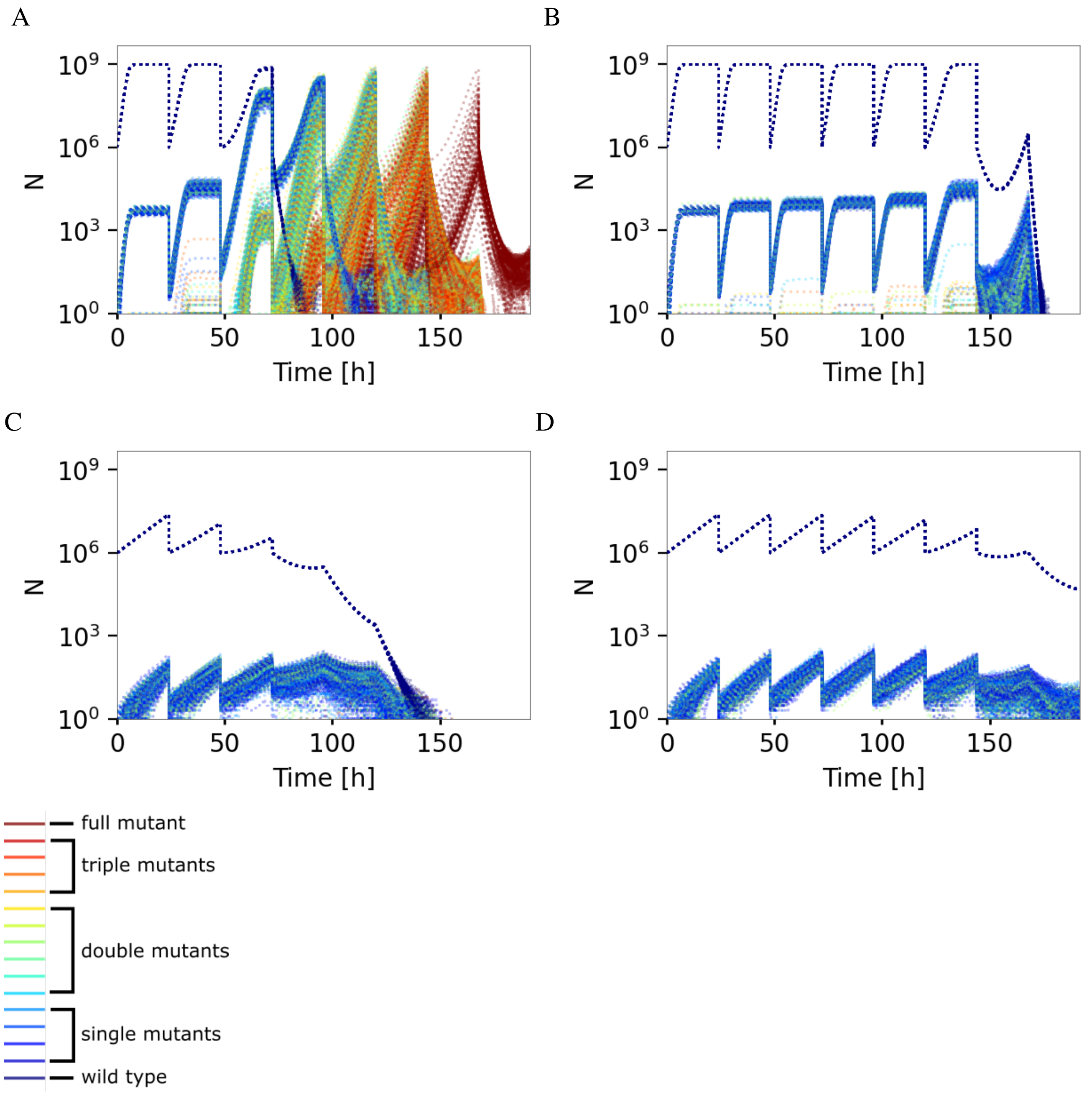

Figure S1. Population dynamics in the passage experiment. A) Only genetic mechanisms considered (i.e. Plankton). B) Biofilm inhabiting bacteria protected by extracellular matrix (reduced penetration). C) Biofilm inhabiting bacteria protected by physiological alterations. D) Both physiological alterations and extracellular matrix considered. Showing 100 stochastic simulations each. Biofilm cost 0.9, biofilm benefit 10.

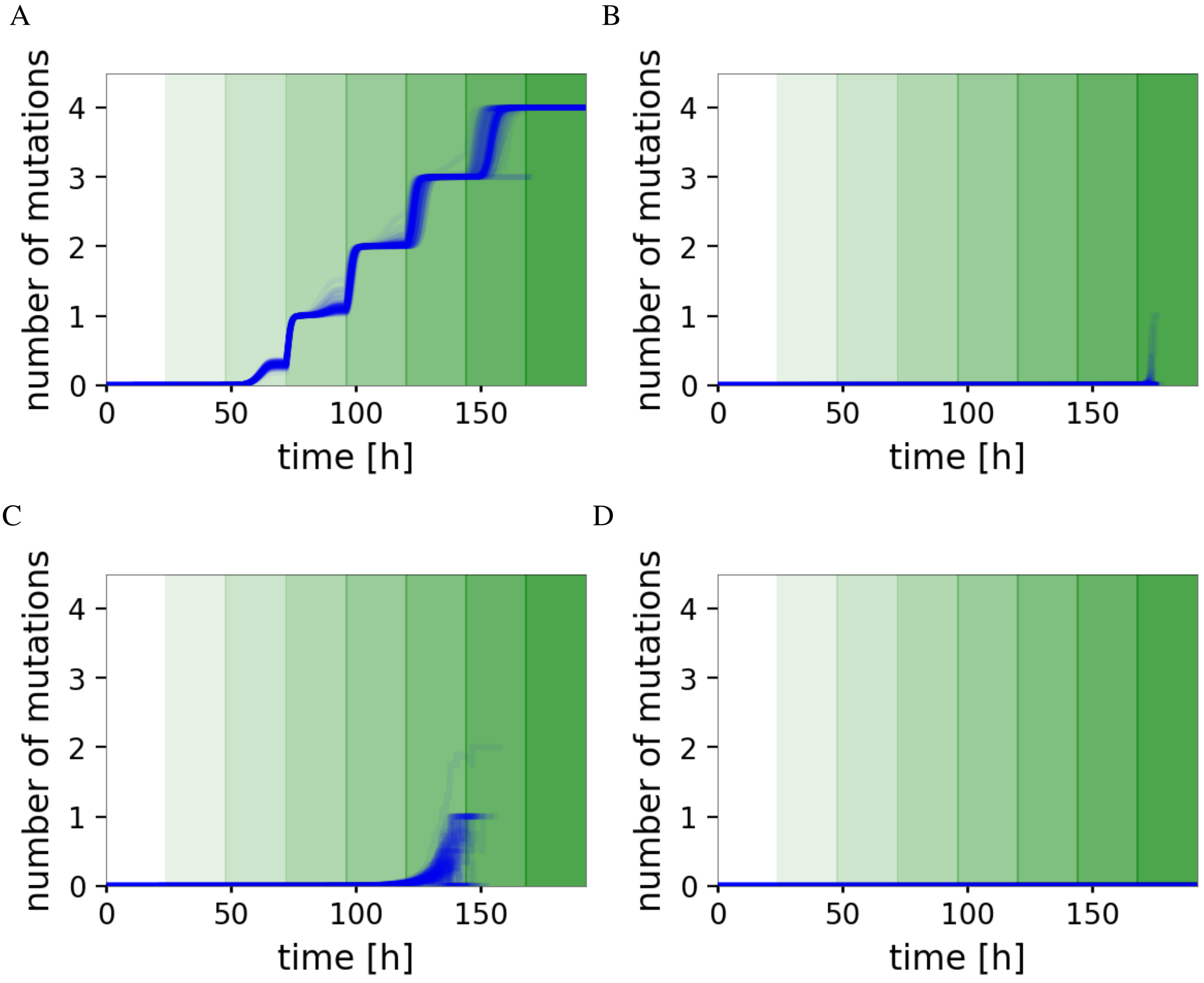

Figure S2. Accumulating number of mutations. The average number of mutations per cell in biofilm. A) Only genetic mechanisms considered (i.e. Plankton). B) Biofilm inhabiting bacteria protected by extracellular matrix (reduced penetration). C) Biofilm inhabiting bacteria protected by physiological alterations. D) Both physiological alterations and extracellular matrix considered. Showing 100 stochastic simulations each. Biofilm cost 0.9, biofilm benefit 10.

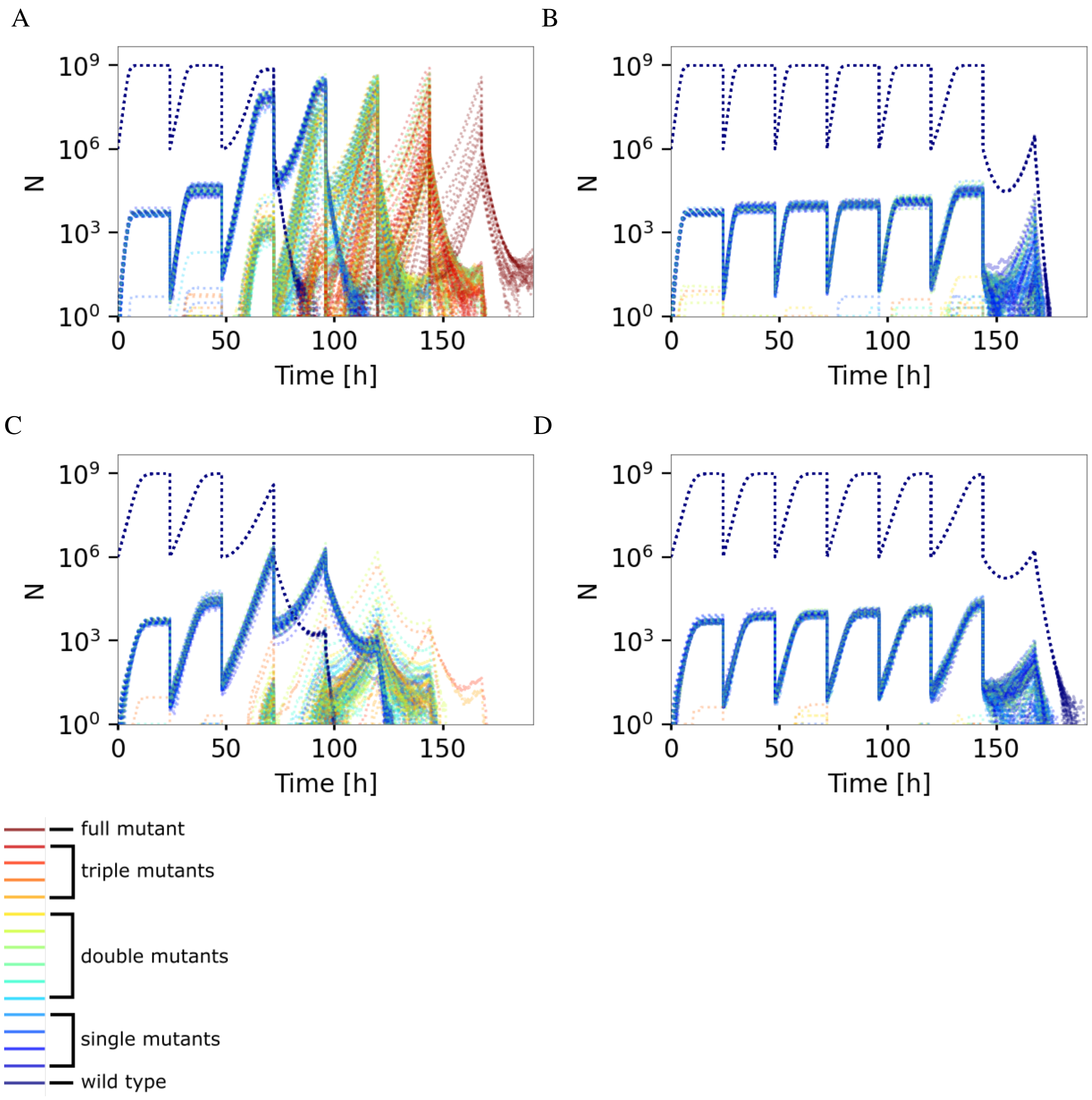

Figure S3. Population dynamics in the passage experiment. A) Only genetic mechanisms considered (i.e. Plankton). B) Biofilm inhabiting bacteria protected by extracellular matrix (reduced penetration). C) Biofilm inhabiting bacteria protected by physiological alterations. D) Both physiological alterations and extracellular matrix considered. Showing 20 stochastic simulations each. Biofilm cost 0.5, biofilm benefit 10.

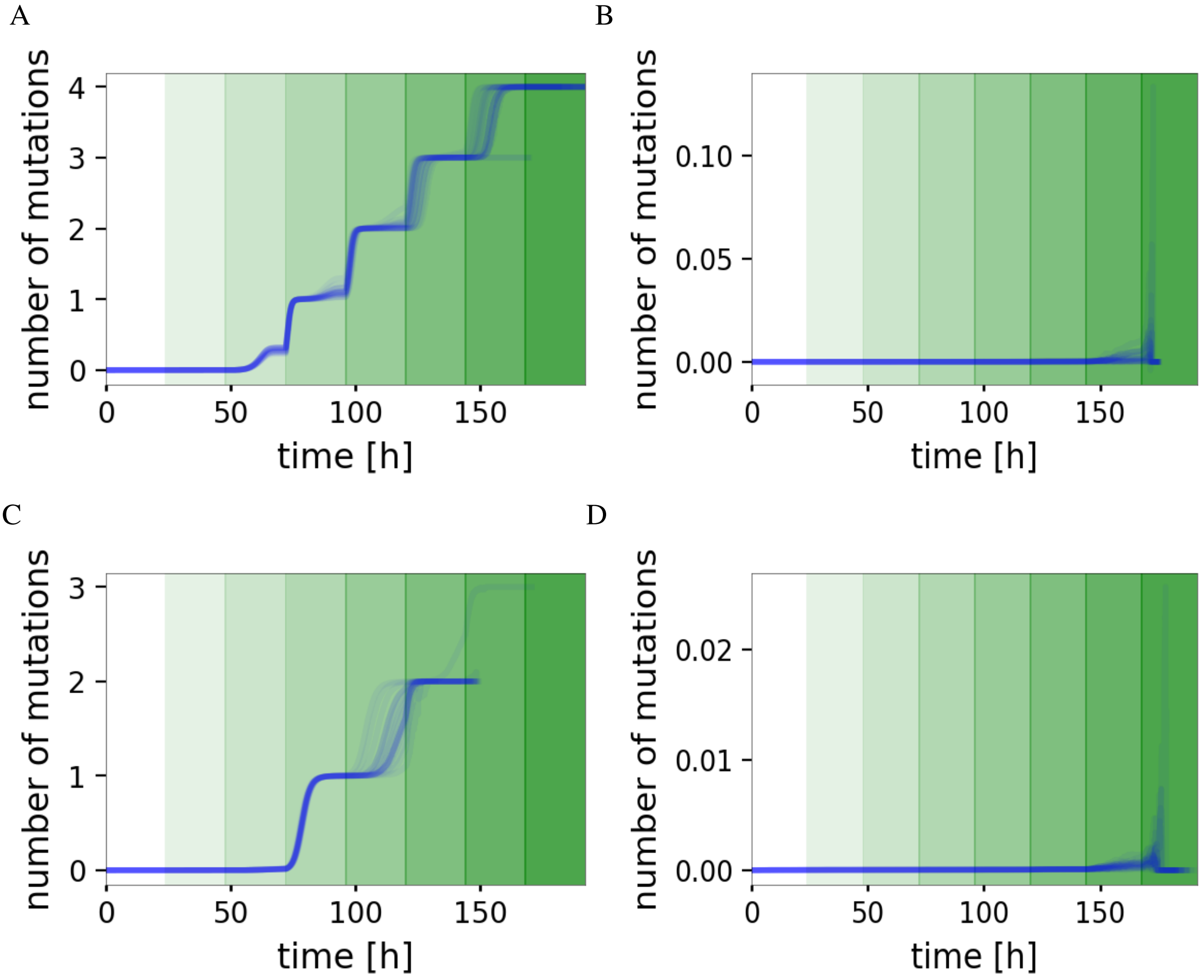

Figure S4. Accumulating number of mutations. A) Only genetic mechanisms considered (i.e. Plankton). B) Biofilm inhabiting bacteria protected by extracellular matrix (reduced penetration). C) Biofilm inhabiting bacteria protected by physiological alterations. D) Both physiological alterations and extracellular matrix considered. Showing 20 stochastic simulations each. Biofilm cost 0.5, biofilm benefit 10.

1.2.2 Facilitating resistance evolution

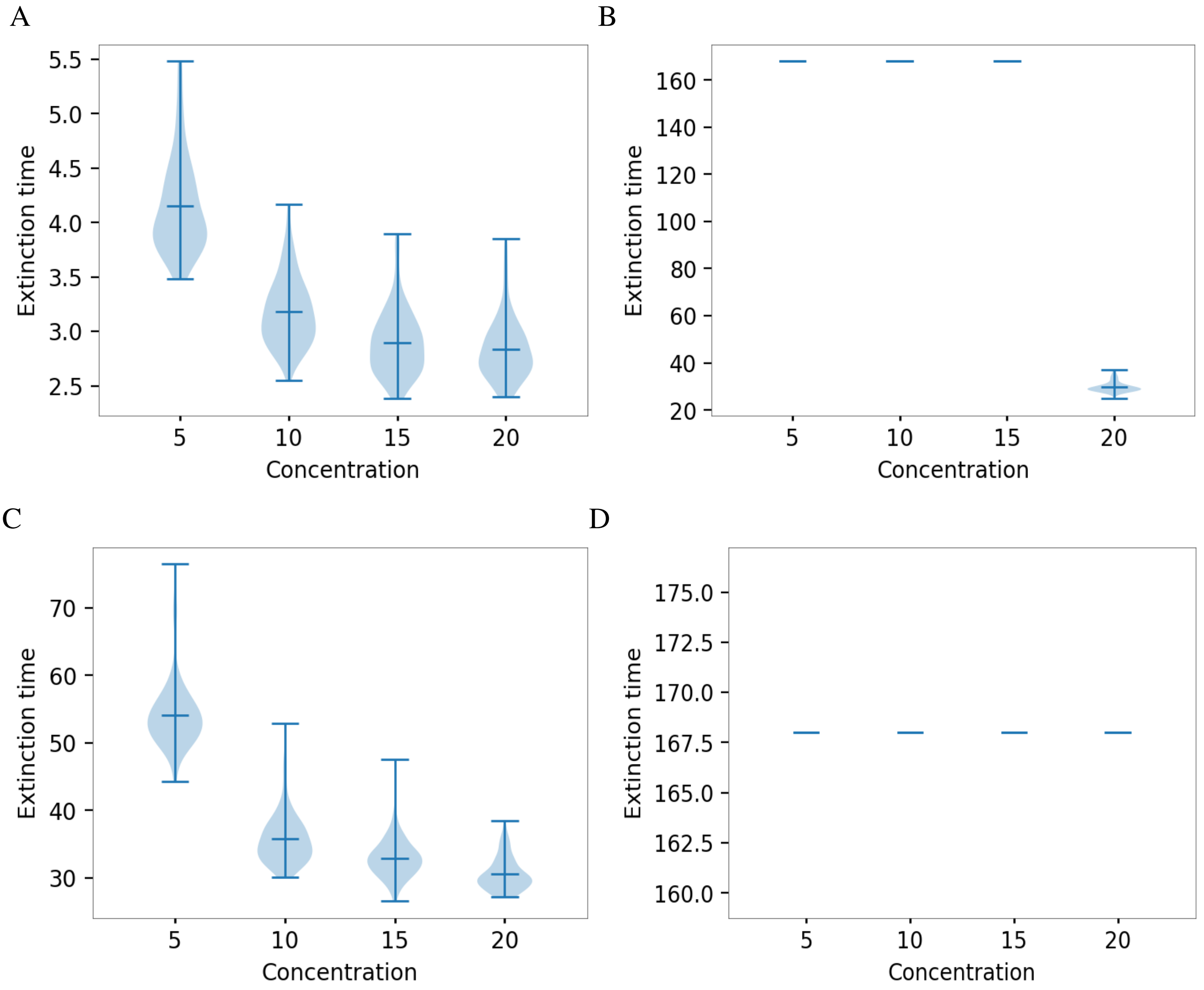

Figure S5. Distribution of extinction times. A) Only genetic mechanisms considered (i.e. Plankton). B) Biofilm inhabiting bacteria protected by extracellular matrix (reduced penetration). C) Biofilm inhabiting bacteria protected by physiological alterations. D) Both physiological alterations and extracellular matrix considered. Biofilm cost 0.9, biofilm benefit 10.

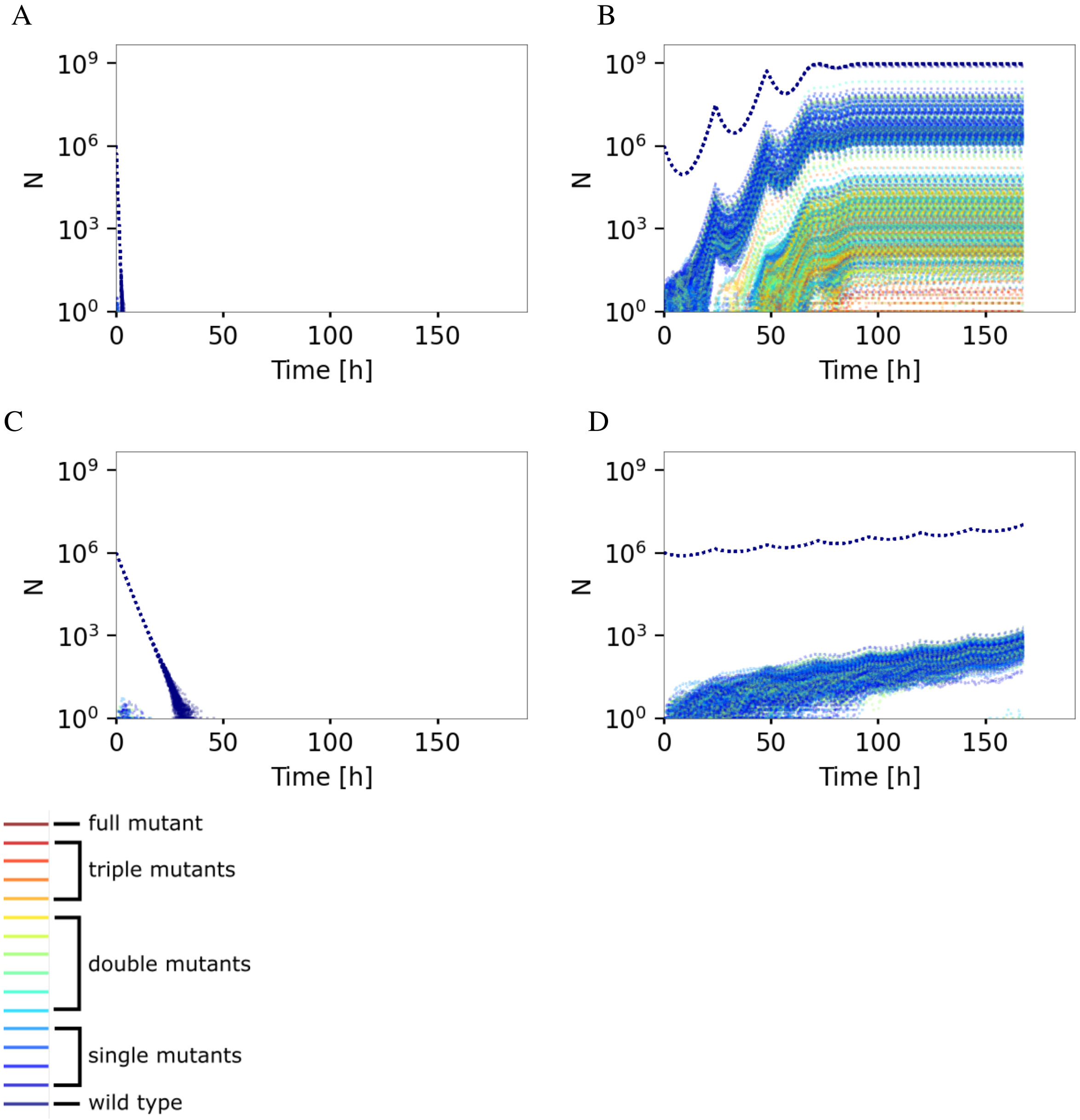

Figure S6. Population dynamics in the repeated treatment, with initial concentration C=15 MIC. A) Only genetic mechanisms considered (i.e. Plankton). B) Biofilm inhabiting bacteria protected by extracellular matrix (reduced penetration). C) Biofilm inhabiting bacteria protected by physiological alterations. D) Both physiological alterations and extracellular matrix considered. Showing 100 stochastic simulations each. Biofilm cost 0.9, biofilm benefit 10.

1.2.3 Interaction between plankton and biofilm

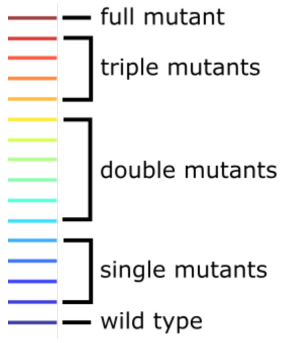

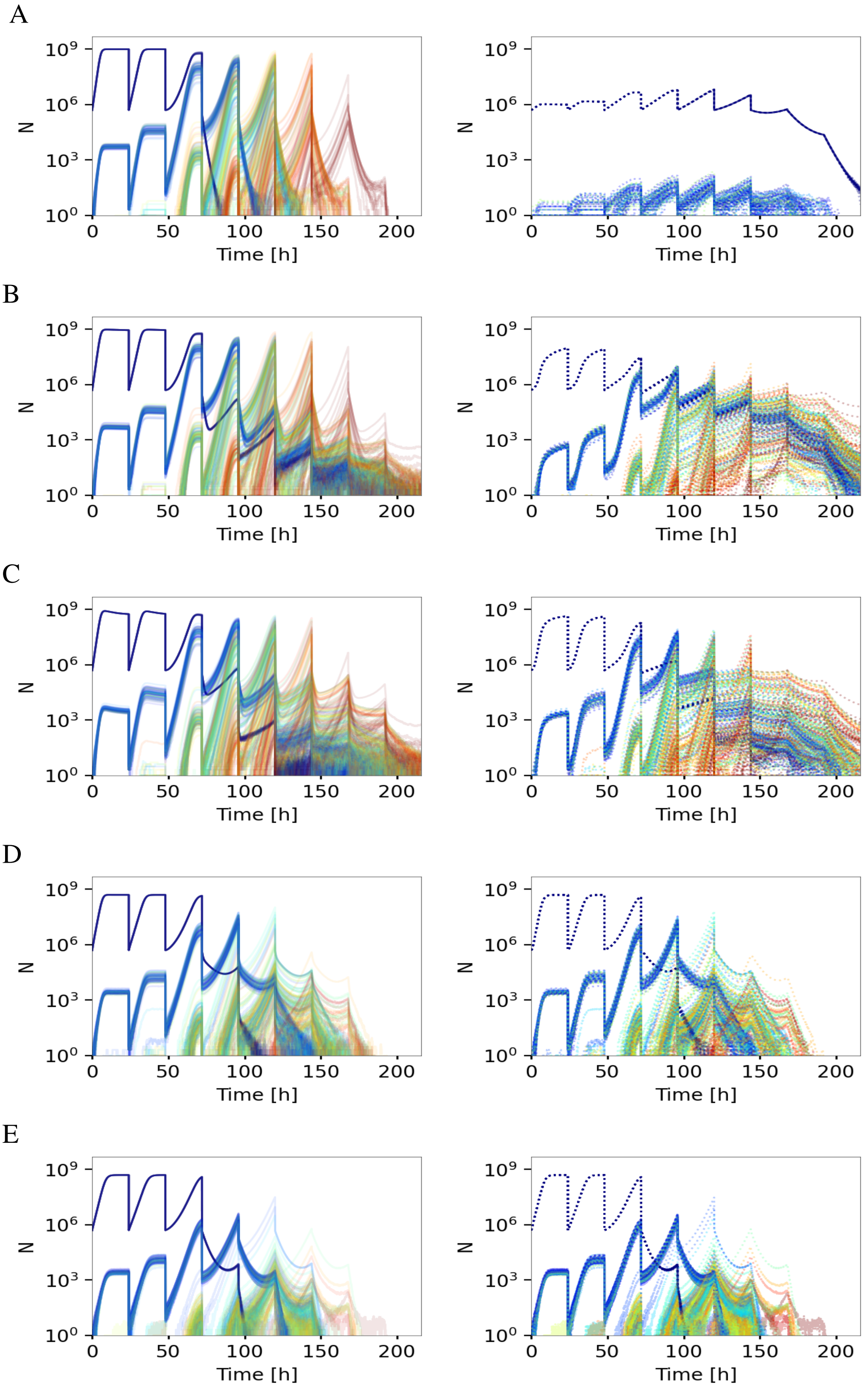

Figure S7. Population dynamics when interactions are considered. Left - plankton, right - biofilm. From top to bottom - increasing interaction (attachment and dispersal rate): A) 0; B) 0,0001; C) 0,001; D) 0,01; E) 0,1.

REFERENCES

Igler, C., Rolff, J., and Regoes, R. (2021). Multi-step vs. single-step resistance evolution under different drugs, pharmacokinetics and treatment regimens. *Elife* 10

Lallemand, E. A., Lacroix, M. Z., Toutain, P.-L., Boullier, S., Ferran, A. A., and Bousquet-Melou, A. (2016). In vitro degradation of antimicrobials during use of broth microdilution method can increase the measured minimal inhibitory and minimal bactericidal concentrations. *Frontiers in Microbiology* 7

Miller, C. R., Monk, J. M., Szubin, R., and Berti, A. D. (2021). Rapid resistance development to three antistaphylococcal therapies in antibiotic-tolerant staphylococcus aureus bacteremia. *PLOS ONE* 16, 1–15. doi:10.1371/journal.pone.0258592

Pan, M. and Chu, L. (2016). Adsorption and degradation of five selected antibiotics in agricultural soil. *Science of The Total Environment* 545-546, 48–56
